# Optimal control for a SIR epidemic model with limited quarantine

**DOI:** 10.1038/s41598-022-16619-z

**Published:** 2022-07-22

**Authors:** Rocío Balderrama, Javier Peressutti, Juan Pablo Pinasco, Federico Vazquez, Constanza Sánchez de la Vega

**Affiliations:** 1 Departamento de Matemática, Facultad de Ciencias Exactas y Naturales, Universidad de Buenos Aires, Ciudad Universitaria, Pabellón I, C1428EGA Buenos Aires, Argentina; 2 Departamento de Física, Instituto de Física de Mar del Plata (IFIMAR) CONICET, UNMDP, Universidad Nacional de Mar del Plata, Funes 3350, 7600 Mar del Plata, Argentina; 3 IMAS-CONICET, Ciudad Universitaria, Pabellón I, C1428EGA Buenos Aires, Argentina; 4 Instituto de Cálculo, FCEN, Universidad de Buenos Aires and CONICET, C1428EGA Buenos Aires, Argentina

**Keywords:** Systems biology, Diseases, Mathematics and computing

## Abstract

Social distance, quarantines and total lock-downs are non-pharmaceutical interventions that policymakers have used to mitigate the spread of the COVID-19 virus. However, these measures could be harmful to societies in terms of social and economic costs, and they can be maintained only for a short period of time. Here we investigate the optimal strategies that minimize the impact of an epidemic, by studying the conditions for an optimal control of a Susceptible-Infected-Recovered model with a limitation on the total duration of the quarantine. The control is done by means of the reproduction number $\sigma (t)$, i.e., the number of secondary infections produced by a primary infection, which can be arbitrarily varied in time over a quarantine period *T* to account for external interventions. We also assume that the most strict quarantine (lower bound of $\sigma$) cannot last for a period longer than a value $\tau$. The aim is to minimize the cumulative number of ever-infected individuals (recovered) and the socioeconomic cost of interventions in the long term, by finding the optimal way to vary $\sigma (t)$. We show that the optimal solution is a single *bang-bang*, i.e., the strict quarantine is turned *on* only once, and is turned *off* after the maximum allowed time $\tau$. Besides, we calculate the optimal time to begin and end the strict quarantine, which depends on *T*, $\tau$ and the initial conditions. We provide rigorous proofs of these results and check that are in perfect agreement with numerical computations.

## Introduction

The Covid-19 pandemic outbreak raises an unprecedented series of decisions in different countries around the world. Since vaccines and effective pharmaceutical treatments were not initially available, governments had decided to impose non-pharmaceutical interventions like social distance, quarantines and total lock-downs as the most effective tools to mitigate the spread of the disease. Although these kinds of measures are helpful in reducing the virus transmission and giving time to health systems to adapt, they could be extremely stressful in terms of economic and social costs, and, in longer periods, tend to have less compliance with the population.

In this article we consider the classical SIR model introduced by Kermack and McKendrick^1^ and widely used in epidemiology^2,3^, where the population is divided in compartments of Susceptible, Infected and Recovered (or Removed) individuals. As it is usual in SIR models, we assume that people who have recovered develop immunity and, therefore, would not be able to get infected nor infect others. We consider that infection and recovery rates are allowed to change over time, and that are homogeneous among the population, instead of heterogeneous rates, not depending on age, individual protection measures, or awareness^4,5^. Let us observe that these heterogeneous rates implies the existence of effective rates, obtained as weighted means of the individual rates^6^. However, for age-structured models, better results are obtained by considering integro-differential equations, and different tools from mathematical control theory are needed^7^. We also assume a mean-field hypothesis that implies random interactions between any pair of agents, unlike other works^8–10^ where interactions are mediated by an underlying network of contacts. In this case, by weighted means of agents degrees, it is possible to derive a system equivalent to a SIR model^11^. Also, a full proof of a derivation using probabilistic tools was studied recently^12^ together with the optimal control problem for vaccination.

Optimal control problems for a system governed by a SIR or a SEIR model (with the addition of the Exposed compartment) with pharmaceutical interventions as vaccination or treatment were widely studied^13–16^. On its part, in the field on optimal control problems, non-pharmaceutical interventions were studied mostly for a control consisting of isolation acting only on the infected^17–20^. Taking into account that there is a window of time when the infected are not detected, in this article we will consider that the quarantine is applied to the whole population. Non-pharmaceutical interventions can range from a mild mitigation policy to a strong suppression policy. As discussed by Ferguson et al.^21^, a suppression policy “*aims to reverse epidemic growth, reducing case numbers to low levels and maintaining that situation indefinitely*”. Suppression can be achieved by restricting travels, closing schools and nonessential businesses, banning social gatherings, and asking citizens to shelter in place. These measures, often referred to as a lockdown, are highly restrictive on social rights and damaging to the economy. In contrast, a mitigation policy “*focuses on slowing but not necessarily stopping epidemic spread*”. Mitigation measures may involve discouraging air travel while encouraging remote working, requiring companies to provide physical separation between workers, banning large gatherings, isolating the vulnerable, and identifying and quarantining contagious individuals and their recent contacts.

A critical parameter in the SIR model is the basic reproduction number $R_0$, defined as the expected number of cases directly generated by one case in a population where all individuals are susceptible to infection. At the beginning of the epidemic, when no one in the population is immune, infected individuals will infect $R_0$ other people on average. Let us observe that, for $R_0<1$, the number of new cases decline, and when $R_0>1$, the number of new cases grows. However, at any time $t>0$, the effective reproduction number $R_t$ replaces $R_0$, since the number of contacts between infected and susceptible agents is reduced due to the interactions with recovered individuals that are immune. Hence, the epidemic grows until a sufficient fraction of the population becomes infected, and after reaching a peak starts to gradually decline. Following Ferguson et al.^21^, for the Covid-19 the suppression phase can achieve $R_0<1$, while the mitigation measures are unlikely to bring $R_0$ below 1. Therefore, the number of new cases are expected to decline during the suppression phase and to start rising again during the mitigation phase, although at a slower rate than in a non-intervention scenario.

In this work we assume that the intervention will occur in a preset period of time *T*, as proposed by Greenhalgh^22^ and recently by Ketcheson^23^, since it is unrealistic that interventions can be sustained indefinitely. Also, the lockdown or (strict quarantine) can last at most for a period $\tau <T$, the maximum time that the population will adhere. Now, there are several interesting questions related to the implementation of the measures:

1. When should the suppression policy begin in [0, *T*]?
2. Is it convenient to split the maximum time $\tau$ into different intervals?
3. Is it better to apply a strong lockdown followed by mild mitigation measures or not?

In this article we study the previous questions using optimal control tools and numerical computations^24–27^. The answers clearly depend on the goal, which in our case is to minimize the overall impact of the epidemics in terms of the final number of infected individuals and the social and economic cost of the interventions, which we assume to increase as the quarantine becomes more strict. To account for quarantine measures, we consider a time-dependent reproduction number. Using an optimal control approach we show that the optimal strategy is of a single *bang-bang* type, that is, the lockdown or strict quarantine is applied in a single interval of time. Moreover, we characterize the time to start and finish the lockdown during the intervention phase. Let us remark that these questions make sense also in SIHR models, which include hospitalized individuals, since it must be necessary to keep the maximum of the hospitalized group below some threshold^28^.

Recently, many works have appeared dealing with these and related issues. The optimal time to start the suppression measures that maximizes this type of objective function was studied by Ketcheson^23^, where it was proved that a bang-bang control is optimal. However, in that work it is assumed that the lockdown corresponds to a zero reproduction number, something that is impossible to achieve in the real world. Moreover, it is assumed that the strict lockdown can last during the whole intervention, which seems to be impracticable. This problem was also analyzed for a different objective function^29^, i.e., minimizing the peak of infected individuals, for which they proved that the optimal policy is not bang-bang. Besides, Kruse and Strack^30^ minimize a functional that depends on the number of infectives during the intervention plus a term that measures the social and economic cost of interventions, and prove that the optimal control is bang-bang, but they do not investigate the optimal time to start the suppression policy.

The second question is suggested by the strategy proposed by Ferguson et al.^21^: the lockdown must be turned *on* and *off* several times based on the incidence of the virus in the population. A control-theoretic approach was considered in several works^28,31–33^, although no time limits for the interventions were imposed. We shall see that the optimal policy is of bang-bang type, which consists on turning the lockdown *on* only once and turning it *off* after the maximum allowed time $\tau$, in agreement with other authors^23,30^.

Finally, the third question involves both suppression and mitigation phases, and one of the policies was colorfully characterized as *the hammer and the dance* in^34^: a strict lockdown, followed by mitigation measures in order to keep under control the propagation of the disease. However, our main results indicate that the best strategy actually depends on the initial condition, determined by the relation between $R_0$ and the initial fraction of susceptible individuals $x_0$. On the one hand, when $x_0$ is smaller than $1/R_0$ the optimal strategy consists on applying a strong lockdown right at the beginning of the intervention period [0, *T*] for the maximum time period $\tau$, followed by a mild mitigation measure until the end of the intervention (strong-mild strategy). On the other hand, when $x_0$ is larger than $1/R_0$ the optimal strategy is to apply mild mitigation measures at the beginning of the intervention, followed by a strong lockdown, and then a mild mitigation measure again in some cases (mild-strong-mild strategy). In this case, the optimal time to start the strong lockdown depends non-trivially on the initial condition.

The paper is organized as follows. We start by describing the basic SIR model in “The SIR model” section, and by introducing the SIR model with control in “The SIR control model” section. We then present the main results of the article supported by numerical simulations in “Results” section, followed by a discussion and conclusions including future work in “Discussion and conclusions” section. Finally, in “Methods” section we provide rigorous proofs of the results by applying Pontryagin’s maximum principle to the control problem, and we prove that the optimal control is bang-bang in Lemma 2. The main results that characterize the optimal control are given in Theorem 2, Corollary 1 and Theorem 3, for different case scenarios.

## The SIR model

**Figure 1:**
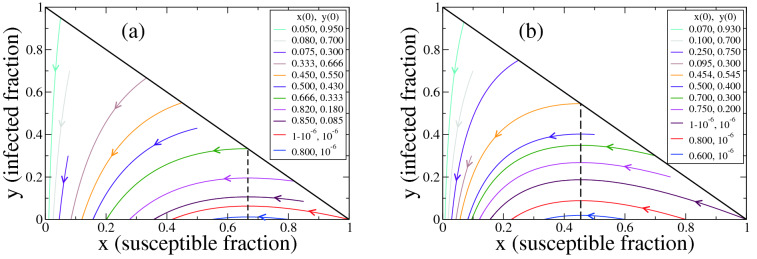
Trajectories of the system in the $x-y$ phase space for the SIR model Eq. (1) with basic reproduction number $\sigma =1.5$ (**a**) and $\sigma =2.2$ (**b**), where *x* and *y* are the fractions of susceptible and infected individuals, respectively. Each curve corresponds to a trajectory starting from a given initial condition (*x*(0), *y*(0)), as indicated in the legends. Arrows denote the direction of the time evolution of *x* and *y*. The vertical dashed line corresponds to the critical value $x=1/\sigma$ where $y'=0$.

The basic SIR compartmental model of infectious diseases introduced by Kermack and McKendrik^1^ considers a population of individuals that is divided in three compartments with homogeneous characteristics: Susceptible (S), Infected (I), and Recovered or Removed (R). The fraction of susceptible, infected and recovered individuals at time *t* is denoted by *x*(*t*), *y*(*t*) and $z(t)=1-x(y)-y(t)$, respectively. It is assumed that each infected individual is in contact with an average number of $\beta$ random individuals per unit time, and that infects only those who are susceptible ($S \rightarrow I$ transition), generating new infections at an average rate $\beta x(t)$. Besides, each infected individual recovers at a rate $\gamma$ ($I \rightarrow R$ transition). Births and deaths are neglected, and the recovered population is assumed to no longer infect others and cannot be reinfected. The infection and recovery rates $\beta$ and $\gamma$, respectively, are related to the basic reproduction number $\sigma$ by $\sigma =\beta /\gamma$, that is, the mean number of infections produced by a single infected individual in a totally susceptible population ($x=1$) during its mean infectious period $1/\gamma$. Then, the evolution of the system is governed by the following set of coupled nonlinear ordinary differential equations (see Hethcote^26^, section 2.1):

1a $$\begin{aligned} x'(t)&= -\gamma \, \sigma \, x(t) \, y(t), \end{aligned}$$

1b $$\begin{aligned} y'(t)&= \gamma \, \sigma \, x(t) \, y(t) - \gamma \, y(t), \end{aligned}$$

with $(x(0),y(0))\in {{\mathscr {D}}}=\left\{ (x_0,y_0): x_0>0, y_0>0, x_0+y_0\le 1\right\}$. Here $x'$ and $y'$ are short notations for the time derivatives *dx*/*dt* and *dy*/*dt*, respectively. The region ${{\mathscr {D}}}$ is forward-invariant and there exists a unique solution for all time^26^. Then, since $y_0>0$, the proportion of infectious individuals is positive at any time. Even though the temporal dynamics of Eq. (1) depends on both $\sigma$ and $\gamma$, the set of system’s trajectories on the $x-y$ space depends only on the basic reproduction number $\sigma$ because $\gamma$ only affects the overall time scale of the system. In Fig. 1 we depict typical trajectories starting from different initial conditions *x*(0), *y*(0), for $\sigma =1.5$ [panel (a)] and $\sigma =2.2$ [panel (b)].

The system of Eq. (1) is at equilibrium if $y(t) = 0$. This equilibrium is stable only if $x(t) \le 1/\sigma$, a condition referred to as herd immunity. If this condition is not satisfied at the initial time ($x(0)>1/\sigma$), then *y*(*t*) first increases until it reaches its maximum value at a time *t* for which $x(t)=1/\sigma$ (dashed vertical lines in Fig. 1), and then decreases and approaches zero asymptotically, i.e., $y_{\infty } \equiv \lim _{t \rightarrow \infty } y(t)= 0$. That is, for $y(0)>0$, $\gamma \ge 0$ and $\sigma \ge 0$ is *y*(*t*) larger than zero for any finite time $t \ge 0$. The fraction of susceptible individuals *x*(*t*) is strictly decreasing, and its value in the long time limit $x_{\infty } \equiv \lim _{t \rightarrow \infty } x(t)$ is always positive. Therefore, the state of the system in the long time limit consists only of susceptible and recovered individuals, $x_{\infty } + z_{\infty } =1$, where $z_{\infty } \equiv \lim _{t\rightarrow \infty } z(t)$. Also, it is known that $x_{\infty } \in (0,1/\sigma )$ (see Theorem 2.1 of the work by Hethcote^26^).

## The SIR control model

We now extend the classical SIR model to address the problem of controlling the spread of an epidemics with no access to vaccination, where the only possible control is isolation. We model this non-pharmaceutical intervention via a time dependent reproduction number $\sigma (t)$ that can be varied in the interval $[\sigma _s,\sigma _m]$, where $\sigma _s$ corresponds to a more strict isolation (“strict” quarantine) than $\sigma _m$ (“mild” quarantine), with $0 \le \sigma _s < \sigma _m$, and assume that this intervention can only be applied over a finite time interval [0, *T*]. Here *T* is the length of the intervention period. After the intervention, the restrictions are removed, thus the disease spreads freely and $\sigma (t)=\sigma _{f} \ge \sigma _m$ for all $t>T$. We think of the control parameter $\sigma (t)$ as capturing political measures such as social distancing, and the lockdown of businesses, schools, universities and other institutions. Then, the system evolves according to the following set of coupled nonlinear ordinary differential equations:

2a $$\begin{aligned} x'(t)&= -\gamma \, \sigma (t) \, x(t) \, y(t), \end{aligned}$$

2b $$\begin{aligned} y'(t)&= \gamma \, \sigma (t) \, x(t) \, y(t) - \gamma \, y(t), \end{aligned}$$

with $(x(0),y(0))\in {{\mathscr {D}}}=\left\{ (x_0,y_0): x_0>0, y_0>0, x_0+y_0\le 1\right\}$, $\sigma (t) \in [\sigma _s,\sigma _m]$ for $t\in [0,T]$ and $\sigma (t)=\sigma _f$ for $t>T$, where $0\le \sigma _s<\sigma _m\le \sigma _f$. We also assume that during the intervention period [0, *T*] it is not possible to impose an extremely restrictive isolation for a long time. Thus, we consider that the strict quarantine—corresponding to $\sigma _s$—can last at most for a fixed time period $\tau$, with $\tau \in (0,T)$. Once the period of intervention is finished at time *T* we compute $x_{\infty }(x(T),y(T),\sigma _f)=\lim _{t\rightarrow \infty } x(t)$, where (*x*(*t*), *y*(*t*)) is the solution of the system of Eq. (2) with initial condition (*x*(*T*), *y*(*T*)) and constant reproduction number $\sigma (t)\equiv \sigma _f$ for $t>T$. Note that in this case, from Hethcote^26^ we deduce that $x_{\infty }(x(T),y(T),\sigma _f)\in (0,1/\sigma _f)$.

While political measures reduce the spread of the disease, they often come at an important economic and social cost. A long and strict quarantine can be very effective at reducing contagions, but at the expense of having a negative impact on the economy. Our goal is to find the optimal control on the SIR model described above that minimizes the total damage of a pandemic in terms of both, the total number of infections and also the socioeconomic costs. We model this trade-off by considering a global cost capturing the total number of individuals that were infected during the epidemics, i.e., those who are recovered in the long-time limit $z_{\infty }$, and the socioeconomic cost of shutting down society during the intervention on [0, *T*], which we assume to increase as $\sigma$ decreases (more restrictions). In order to find the optimal $\sigma (t)$ it proves convenient to work with the fraction of susceptible individuals in the long term $x_{\infty }$ instead. Then, given that minimizing $z_{\infty }$ is equivalent to maximizing $x_{\infty }$, since $x_{\infty }+z_{\infty }=1$, we define the functional

3 $$\begin{aligned} J(x,y,\sigma ):=x_{\infty }(x(T),y(T),\sigma _f) + C(\sigma ), \end{aligned}$$

where

4 $$\begin{aligned} C(\sigma ) := \int _0^T L(\sigma (t)) dt. \end{aligned}$$

Our goal is to maximize the functional *J*, which has the following interpretation. The first term of *J* is the fraction of individuals that remain susceptible in the long term $x_{\infty }$, and that we want to keep as large as possible subject to the condition of maximizing the second term of *J* as well, the functional $C(\sigma )$. The functional $C(\sigma )$ is taken to be inversely proportional to the socioeconomic cost of the intervention ($C(\sigma )$ increases as the cost decreases), as the function *L* is assumed to be a monotonously increasing function of $\sigma$. Then, an increase of the socioeconomic cost is achieved by decreasing $\sigma$ (more restrictions or stricter quarantine), which leads to decreasing *L* and consequently $C(\sigma )$. Therefore, we see that there is a non-trivial competition between the two terms of Eq. (3), given that by decreasing $\sigma$ the value of $x_{\infty }$ increases, while $C(\sigma )$ decreases.

In the next section we describe the main results about the optimal control and we test them via numerical simulations.

## Results

As mentioned in the last section, the optimal control is given by the shape of $\sigma (t)$ that minimizes both, the final number of infected individuals and the socioeconomic costs, which corresponds to maximizing the functional *J* from Eqs. (3) and (4). From now on we restrict ourselves to the case where the socioeconomic cost of imposing a quarantine is linear in the control $\sigma (t)$. This is a simplified first approach that narrows the analysis of the general problem formulated in ”The SIR control model” section but, as we shall see, has the advantage of providing further insight into the structure of the optimal policy. The assumption that the cost of socioeconomic measures that reduce the transmission rate is linear in $\sigma$ can be interpreted in the context of social distancing as given by Kruse and Strack^30^: “Shutting down half of the economy for two days is equally costly as shutting down the whole economy for a single day”. Then, we consider that the function *L* in Eq. (4) is a linear and increasing function that depends only on the control $\sigma$, that is $L(\sigma (t))=\kappa \sigma (t)$, which satisfies the condition of being a monotonically increasing function of $\sigma$ expressed in the last section. In this case, the parameter $\kappa \ge 0$ could be interpreted as the assessment that a policy maker gives to the socioeconomic impact of the quarantine compared to the final number of infected individuals. In this regard, $\kappa$ is a fixed real number that can be chosen small enough by a government that intends to reduce the final number of infected individuals regardless the socioeconomic impact, or can be chosen large enough by a government that can face a large number of final infected and intends to control the socioeconomic impact. Here we mainly focus on the case where $\kappa$ is small (see condition Eq. (33) in “Methods” section). Also, when $\kappa$ is large enough we prove that the optimal strategy consists in calling off the lockdown and take mild mitigation measures for all the intervention period (see Lemma 6 in “Methods” section).

Under these conditions, we prove in “Methods” section that the optimal control is of the form of a single *bang-bang*. This consists on turning the strict quarantine *on* only once and switching it *off* after the maximum allowed time $\tau$ or, eventually, when the intervention ends at time *T*, depending on the initial condition and the values of the strict and mild reproduction numbers $\sigma _s$ and $\sigma _m$, respectively. Then, the problem is reduced to find the optimal time to start the strict quarantine, which we call $t^*$, and its length called $\eta ^*$. We also show in “Methods” section that the length of the strict quarantine $\eta ^*$ for the optimal control could be less than the maximum time $\tau$ in some cases, as we shall see below. This means that, surprisingly, sometimes it is more convenient to make a shorter use of the strict quarantine to obtain better results in terms of pandemic costs.

We analyze the optimal control in three different case scenarios: i) $\kappa =0$, $\sigma _m = \sigma _f$ and $\sigma _s= 0$, ii) $\kappa =0$, $\sigma _m = \sigma _f$ and $\sigma _s>0$ and iii) $\kappa >0$ and $0< \sigma _s< \sigma _m < \sigma _f$. The optimal times $t^*$ and $\eta ^*$ for each case are given in Corollary 1, Theorems 2 and 3, respectively, of “Methods” section, where the interested reader can find rigorous proofs. The optimal control for the different cases, given by $t^*$ and $\eta ^*$, is summarized and numerically tested below for specific parameter values. For that, we integrate the system of Eq. (2) using the Adams’ method^35,36^, for various time periods $\tau$ of the strict quarantine ($\sigma =\sigma _s$) in the bang-bang control, starting at time *t* but ending before *T*, which is the control period ($\sigma (t)=\sigma _f$ for $t > T$). That is, the value of $\sigma (t)$ adopts the following form, depending on *t*, $\tau$ and *T*:

5 $$\begin{aligned} \sigma (r) = {\left\{ \begin{array}{ll} \sigma _m &{} \text{ for } 0 \le r< t, \\ \sigma _s &{} \text{ for } t \le r< t + \eta , \\ \sigma _m &{} \text{ for } t+\eta \le r < T, \\ \sigma _f &{} \text{ for } r \ge T, \end{array}\right. } \end{aligned}$$

where

6 $$\begin{aligned} \eta = {\left\{ \begin{array}{ll} \tau &{} \text{ for } t+\tau \le T ~ \text{ and } \\ T-t &{} \text{ for } t+\tau > T. \end{array}\right. } \end{aligned}$$

We start by testing the simplest case $\kappa =0$, $\sigma _m=\sigma _f$ and $\sigma _s \ge 0$, and we then test the most general case $\kappa >0$ and $0< \sigma _s< \sigma _m < \sigma _f$. We take the value $\gamma =0.1/\text{day }$ for the recovery rate, which corresponds to a mean recovery time of 10 days that falls within the range of Covid-19 estimates^23^. This value of $\gamma$ sets the time scales of the system. For now on all time scales are given in units of “days”, even though we omit units for the sake of simplicity.

### Case $\kappa =0$, $\sigma _m = \sigma _f$ and $\sigma _s= 0$ (Corollary 1)

**Figure 2:**
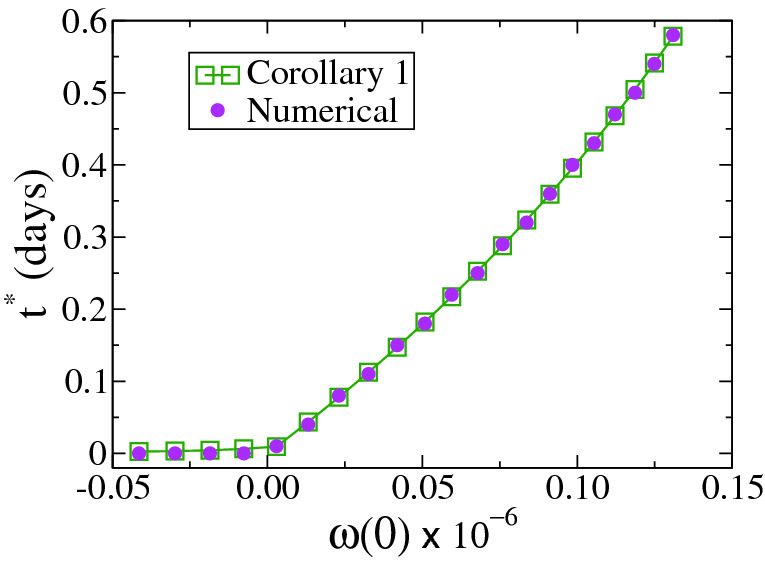
Optimal initial time of the strict quarantine $t^*$ vs *w*(0) for $\tau =10$ and $\kappa =0$. The other parameters are $\gamma =0.1, \sigma _m=\sigma _f=1.5, \sigma _s=0$ and $T=260$.

We first analyze the case $\kappa =0$ with a bang-bang control in the interval [0, *T*] that consists of a mild quarantine $(\sigma _m=1.5)$ and an extremely strict and unrealistic quarantine ($\sigma _s=0$) during which there are no infections. The other parameter in the simulations is $T=260$, together with the initial condition $y_0=y(t=0)=10^{-6}$ and $x_0=x(t=0)=1-10^{-6}$. We can see from Corollary 1 that the optimum initial time of the strict quarantine is $t^*=0$ for $x_0 \le 1/\sigma _f$ ($w(0) \le 0$), while for $x_0 > 1/\sigma _f$ ($w(0)>0$) is given by

7 $$\begin{aligned} t^* = {\left\{ \begin{array}{ll} {\overline{t}} &{} \text{ for } 0 \le \tau \le {\overline{\tau }}, \text{ where } x_{T-\tau ,\tau }(T-\tau ) \le \dfrac{1}{\sigma _f} \text{ and } \eta =\tau , \\ T-\tau &{} \text{ for } {\overline{\tau }}< \tau< {\tilde{\tau }}, \text{ where } \dfrac{1}{\sigma _f} < x_{T-\tau ,\tau }(T-\tau ) \le \frac{1}{\sigma _f(1-e^{-\gamma \tau })} and \eta =T-\tau , \\ {\tilde{t}} &{} \text{ for } \tau \ge {\tilde{\tau }}, \text{ where } x_{T-\tau ,\tau }(T-\tau ) > \frac{1}{\sigma _f(1-e^{-\gamma \tau })}, \text{ and } \eta =T-{\tilde{t}}. \\ \end{array}\right. } \end{aligned}$$

where ${\overline{t}} = T-{\overline{\tau }} \in [0,T-\tau ]$ is the unique value, independent from $\tau \in [0,{\overline{\tau }}]$, such that $x_{{\overline{t}},\tau }({\overline{t}})=\frac{1}{\sigma _f}$. Likewise, ${\tilde{t}}=T-{\tilde{\tau }} \in [T-\tau ,T]$ is the unique value, independent from $\tau \in [{\tilde{\tau }},T]$, such that $x_{{\tilde{t}},T-{\tilde{t}}}({\tilde{t}})= \frac{1}{\sigma _f(1-e^{-\gamma (T-{\tilde{t}})})}$. As $\kappa =0$, $x_{\infty }$ reaches a maximum value when the strict quarantine starts at the optimal time $t^*$ [see Eq. (12)]. In Fig. 2 we plot $t^*$ vs *w*(0) for $\tau =10$, calculated from Eq. (54) (squares) and by estimating the maximum of $x_{\infty }$ (circles). We can see that $t^*$ takes values close to zero for $w(0) \le 0$. In the rest of this section we consider the case $w(0)>0$.

**Figure 3:**
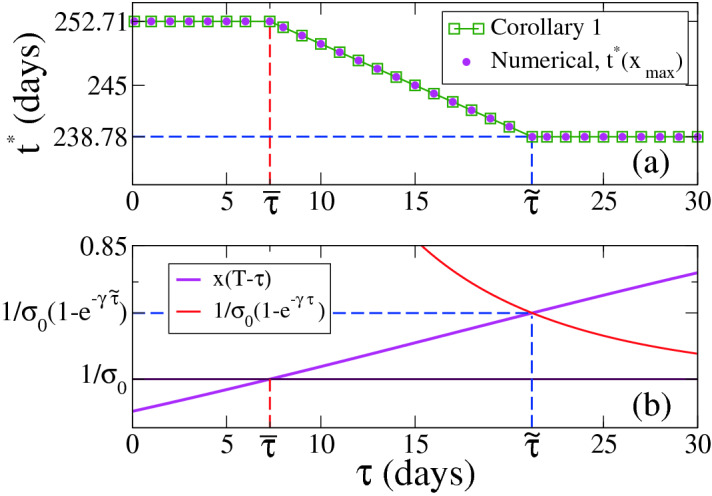
(**a**) Optimal initial time $t^*$ vs strict quarantine length $\tau$ for $\kappa =0$ and $x_0>1/\sigma _f$. (**b**) Graphical determination of the times ${\overline{\tau }}$ and ${\tilde{\tau }}$ that define the three regions for the different behaviours of $t^*$. The parameters are $\gamma =0.1, \sigma _m=\sigma _f=1.5, \sigma _s=0$ and $T=260$. The initial condition corresponds to $x(0)=1-10^{-6}, y(0)=10^{-6}$ ($R_0=\sigma _f x(0) >1$). The optimum times of the first and last regions are ${\overline{t}} \simeq 252.71$ and ${\tilde{t}} \simeq 238.78$, respectively, determined by ${\overline{\tau }} \simeq 7.29$ and ${\tilde{\tau }} \simeq 21.22$.

The behaviour of $t^*$ from Eq. (7) for $x_0>1/\sigma _f$ ($w(0)>0$) is tested in Fig. 3a, where we compare numerical results (circles) with that obtained from Eq. (7) (squares, Corollary 1). We observe that the agreement between numerical computations and Corollary 1 is very good. Figure 3b is an auxiliary plot that shows how to obtain graphically the optimum times ${\overline{t}} \simeq 252.71$ and ${\tilde{t}} \simeq 238.78$ that define the three different regimes of $t^*$ from Eq. (7). These times are obtained by estimating the values of $\tau$ for which the curve $x_{t,T-t}(T-\tau )$ crosses the lines $1/\sigma _f$ and $1/\left[ \sigma _f(1-e^{-\gamma \tau }) \right]$, which happens at ${\overline{\tau }} \simeq 7.29$ and ${\tilde{\tau }} \simeq 21.22$, respectively.

#### *Remark 1*

The effective reproductive number $R_t^{\sigma } \equiv \sigma \, x_{\sigma }(t)$ represents the mean number of individuals that an agent infects during its infectious period, at time *t*. It is interesting to note that the optimal time from Eq. (7) can be rewritten in terms of $R_t^{\sigma }$ as

8 $$\begin{aligned} t^* = {\left\{ \begin{array}{ll} {\overline{t}} &{} \text{ for } 0 \le \tau \le {\overline{\tau }}, \text {where} \; R_{T-\tau }^{\sigma _f} \le 1 \;  \text {and}  \; \eta =\tau , \\ T-\tau &{} \text{ for } {\overline{\tau }} \le \tau \le {\tilde{\tau }}, \text {where} \; 1 < R_{T-\tau }^{\sigma _f} \le \frac{1}{1-e^{-\gamma \tau }}  \; \text {and}  \; \eta =T-\tau , \\ {\tilde{t}} &{} \text{ for } \tau> {\tilde{\tau }}, \text {where} \; R_{T-\tau }^{\sigma _f} > \frac{1}{1-e^{-\gamma \tau }},  \; \text {and}  \; \eta =T-{\tilde{t}}. \\ \end{array}\right. } \end{aligned}$$

Here $R_{T-\tau }^{\sigma _f}=\sigma _f \, x_{\sigma _f}(T-\tau )$, ${\overline{\tau }}=T-{\overline{t}}$ and ${\tilde{\tau }}=T-{\tilde{t}}$, where ${\overline{t}}$ and ${\tilde{t}}$ are determined from the relations

9 $$\begin{aligned} R_{{\overline{t}}}^{\sigma _f} = 1 ~~~~~ \text{ and } ~~~~~ R_{{\tilde{t}}}^{\sigma _f} = \frac{1}{1-e^{-\gamma (T-{\tilde{t}})}}. \end{aligned}$$

**Figure 4:**
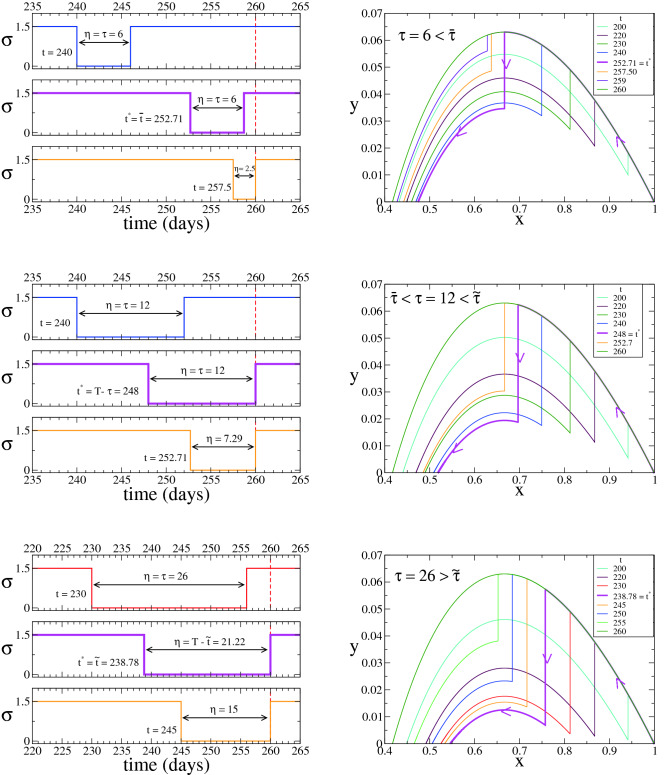
Case $\kappa =0$, $\sigma _m = \sigma _f$ and $\sigma _s= 0$ (Corollary 1). System’s trajectory in the $x-y$ phase space (right panels), for $\gamma =0.1$, $\kappa =0$, $\sigma _m=\sigma _f=1.5$, $\sigma _s=0$ and $T=260$, and the three values of $\tau$ indicated in the legends corresponding to the different regimes of the optimum time $t^*$ (pink lines). Left panels show the time evolution of $\sigma$ for three different initial times *t* of the strict quarantine in each case. The optimum times are $t^*={\overline{t}} \simeq 252.71$ for $\tau =6$ (top panels), $t^*=T-\tau =248$ for $\tau =12$ (middle panels) and $t^*={\tilde{t}} \simeq 238.78$ for $\tau =26$ (bottom panels).

In Fig. 4 we show the evolution of the system in the $x-y$ phase space for a given $\tau$ and various *t* (right panels), together with the evolution of $\sigma (t)$ (left panels), which describe the three different behaviours of $t^*$. All curves start at $(x_0,y_0)=(1-10^{-6},10^{-6})$ and follow the top curve with mild quarantine ($\sigma =\sigma _f$) until the strict quarantine starts at *t* ($\sigma =\sigma _s=0$), vertically falling down up to a lower level curve when the mild quarantine starts, and finally following this curve until the fixed point $(x_{\infty },0)$ is asymptotically reached. The vertical trajectory describes the evolution within the strict quarantine where *x*(*t*) remains constant, given that $\sigma (t)=\sigma _s=0$ in that period. The optimum time $t^*$ that leads to the maximum of $x_{\infty }$ corresponds to the time for which *y*(*t*) drops to the lowest level curve in the interval $[t,t+\eta ]$ (pink curve). For $\tau =6 < 7.29 = {\overline{\tau }}$ (Fig. 4 top panels) we see that the maximum of $x_{\infty }$ is reached starting the strict quarantine at $t^*={\overline{t}}=252.71$, where the effective reproduction number is $R_{{\overline{t}}}^{\sigma _f}=R_{{\overline{t}}+\eta }^{\sigma _f}=1$, and thus there is no new outbreak when the strict quarantine is released ($\frac{dy}{dt} |_{t+\eta }=0$). In this case the entire quarantine period $\eta =\tau$ is used. For ${\overline{\tau }}< \tau =12 < {\tilde{\tau }}=21.22$ (Fig. 4 middle panels) the optimum initial time is $t^*=T-\tau =248 < {\overline{t}}$, obtained by still using the entire strict quarantine period but starting earlier than ${\overline{t}}$. Finally, for $\tau =26 > {\tilde{\tau }}$ (Fig. 4 bottom panels) the optimum is $t^*={\tilde{t}}=238.78>T-\tau =234$, where it turns more effective to use the strict quarantine for a shorter time $T-t^*< \tau$. Notice that implementing a shorter but later strict quarantine is more efficient than using a longer and earlier strict quarantine, as we can see by comparing $\sigma (t)$ for $\eta =26$ and $\eta =21.22$ in the bottom panels for the $\tau =26$ case.

### Case $\kappa =0$, $\sigma _m = \sigma _f$ and $\sigma _s>0$ (Theorem 2)

We now analyze the case $\kappa =0$, $\sigma _m=\sigma _f=1.5$ and $\sigma _s=0.3>0$, with $T=260$. This corresponds to a strict quarantine that is softer than in the previous case $\sigma _s=0$, and during which there are infections. Initially is $y_0=10^{-6}$ and $x_0=1-10^{-6}$. We can see from Theorem 2 that the optimum initial time of the strict quarantine $t^*$ for $w(0)>0$ is

10 $$\begin{aligned} t^* = {\left\{ \begin{array}{ll} {\overline{t}} &{} \text{ for } 0 \le \tau \le {\overline{\tau }}, \text{ where } w(0)\ge 0, w(T-\tau ) \le 0 \; and \;\eta =\tau , \\ T-\tau &{} \text{ for } {\overline{\tau }} \le \tau \le {\tilde{\tau }}, \text{ where } 0 < w(T-\tau ) \le \frac{1}{\gamma y_{T-\tau ,\tau }(T-\tau )} \; \text{ and } \; \eta =T-\tau , \\ {\tilde{t}} &{} \text{ for } \tau> {\tilde{\tau }}, \text{ where } w(T-\tau ) >\frac{1}{\gamma y_{T-\tau ,\tau }(T-\tau )}, \;\text{ and } \;\eta =T-{\tilde{t}}, \\ \end{array}\right. } \end{aligned}$$

where ${\overline{t}}\in [0,T-\tau ]$ is the unique value, depending on $\tau \in [0,{\overline{\tau }}]$, such that $w({\overline{t}})=0$. On the other hand, ${\tilde{t}}\in [T-\tau ,T]$ is the unique value, independent from $\tau \in [{\tilde{\tau }},T]$, such that $w({\tilde{t}})= \frac{1}{\gamma y_{{\tilde{t}},T-{\tilde{t}}}({\tilde{t}})}$. The dependence and independence of *w*(*t*) on $\tau$ for $t\in [0,T-\tau ]$ and $t\in [T-\tau ,T]$, respectively, can be seen from the definition of *w*(*t*) in Eq. (42).

**Figure 5:**
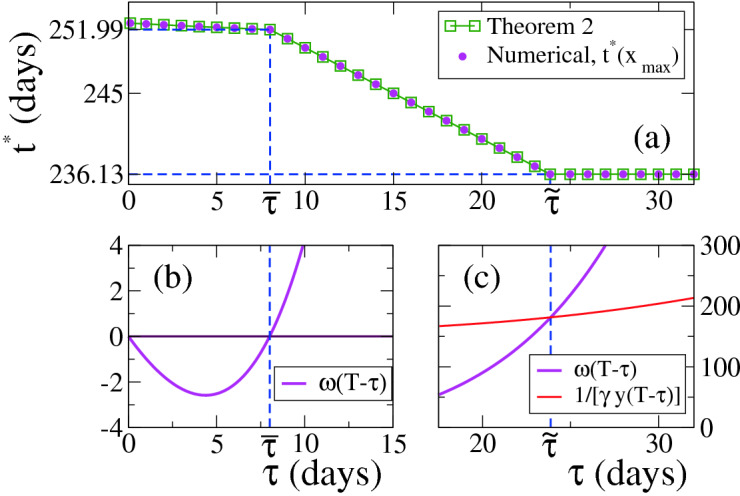
(**a**) Optimal initial time $t^*$ vs strict quarantine length $\tau$ for $\kappa =0$ and $w(0)>0$. (**b**) and (**c**) Graphical determination of the times ${\overline{\tau }}$ and ${\tilde{\tau }}$, respectively, which define the three regions for the different behaviours of $t^*$. The parameters are $\gamma =0.1, \sigma _f=1.5, \sigma _s=0.3, T=260$. The initial condition corresponds to $x(0)=1-10^{-6}, y(0)=10^{-6}$. The optimum time for the region $\tau > {\tilde{\tau }} \simeq 23.87$ is ${\tilde{t}} \simeq 236.13$, while $t^*$ has a slight dependence on $\tau$ for $\tau \le {\overline{\tau }} \simeq 8.01$.

In Fig. 5a we compare numerical results (circles) with results from Eq. (10) (squares, Theorem 2), where we see a very good agreement. At $t^*$, $x_{\infty }$ reaches a maximum. Unlike the $\sigma _s=0$ case, for $\sigma _s=0.3>0$ the optimal time $t^*$ in the $0 \le \tau \le {\overline{\tau }} \simeq 8.01$ interval depends on $\tau$, that is, $t^*={\overline{t}}(\tau )$, while for $\tau > {\tilde{\tau }} \simeq 23.87$ is $t^*={\tilde{t}} \simeq 236.13$ independent of $\tau$. Figure 5b,c show that the optimal times ${\overline{t}}$ and ${\tilde{t}}$ are estimated, respectively, as the values of $t=T-\tau$ for which the curve $w(T-\tau )$ crosses the horizontal line 0 and the curve $1/\left[ \gamma y_{t,T-t}(T-\tau ) \right]$.

**Figure 6:**
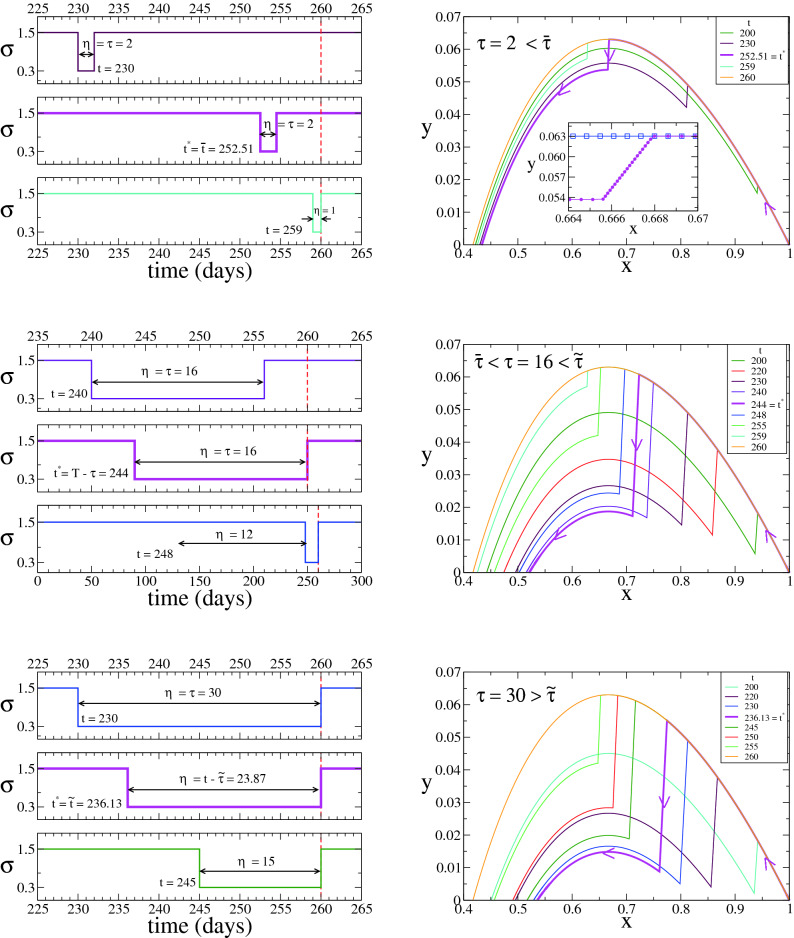
Case $\kappa =0$, $\sigma _m = \sigma _f$ and $\sigma _s>0$ (Theorem 2). System’s trajectory in the $x-y$ phase space (right panels), for $\gamma =0.1$, $\kappa =0$, $\sigma _m=\sigma _f=1.5$, $\sigma _s=0.3$ and $T=260$, and the three values of $\tau$ indicated in the legends corresponding to the different regimes of the optimum time $t^*$ (pink lines). Left panels show the time evolution of $\sigma$ for three different initial times *t* of the strict quarantine in each case. The optimum times are $t^*={\overline{t}} \simeq 252.51$ for $\tau =2$ (top panels), $t^*=T-\tau =244$ for $\tau =16$ (middle panels) and $t^*={\tilde{t}} \simeq 236.13$ for $\tau =30$ (bottom panels).

Figure 6 is analogous to Fig. 4 for the $\sigma _s=0$ case, and depicts the three different behaviours of $t^*$. Curves are similar to those of $\sigma _s=0$, where the main difference is that for $\sigma _s=0.3>0$ the trajectory of the system within the strict quarantine in the $x-y$ space is described by a diagonal line (see inset of top-right panel), given that $\sigma _s$ is larger than zero and thus *x*(*t*) decreases in this period. At the optimum time $t^*$, *y*(*t*) drops to the lowest level curve in the interval $[t,t+\eta ]$ (pink curves).

### General case $\kappa >0$ and $0< \sigma _s< \sigma _m < \sigma _f$ (Theorem 3)

In this section we analyze the most general case $\kappa =10^{-5}>0$, with a mild quarantine ($\sigma _m=1.5$) together with a strict quarantine ($\sigma _s=0.3 < \sigma _m$) during the control interval $t \in [0,T]$, and with $\sigma (t)=\sigma _f=2.2 > \sigma _m$ for the case of no restrictions after the control period $t>T$. We take $T=320$, and the rest of the parameters are the same as those in the previous studied cases. Then, from Theorem 3 the optimum initial time $t^*$ is given by

11 $$\begin{aligned} t^* = {\left\{ \begin{array}{ll} {\overline{t}} &{} \text{ for } 0 \le \tau \le {\overline{\tau }}, \; \text {where} \; w(0)\ge 0, w(T-\tau ) \le 0 \text \; {and} \; \eta =\tau , \\ T-\tau &{} \text{ for } {\overline{\tau }} \le \tau \le {\tilde{\tau }}, \; \text {where} \; 0 < w(T-\tau ) \le \alpha (T-\tau ) \; \text {and} \; \eta =T-\tau , \\ {\tilde{t}} &{} \text{ for } \tau> {\tilde{\tau }}, \;\text {where} \;w(T-\tau ) >\alpha (T-\tau ), \;\text {and} \;\eta =T-{\tilde{t}}, \\ \end{array}\right. } \end{aligned}$$

where ${\overline{t}}\in [0,T-\tau ]$ is a unique value that depends on $\tau \in [0,{\overline{\tau }}]$ and satisfies $w({\overline{t}})=0$, while ${\tilde{t}}\in [T-\tau ,T]$ is a unique value independent of $\tau \in [{\tilde{\tau }},T]$ that satisfies $w({\tilde{t}})= \alpha ({\tilde{t}})$. Here $\alpha (t)$ is given by Eq. (36), whereas the dependence and independence of *w*(*t*) on $\tau$ for $t\in [0,T-\tau ]$ and $t\in [T-\tau ,T]$, respectively, is seen in the definition of *w*(*t*) in Eq. (35).

**Figure 7:**
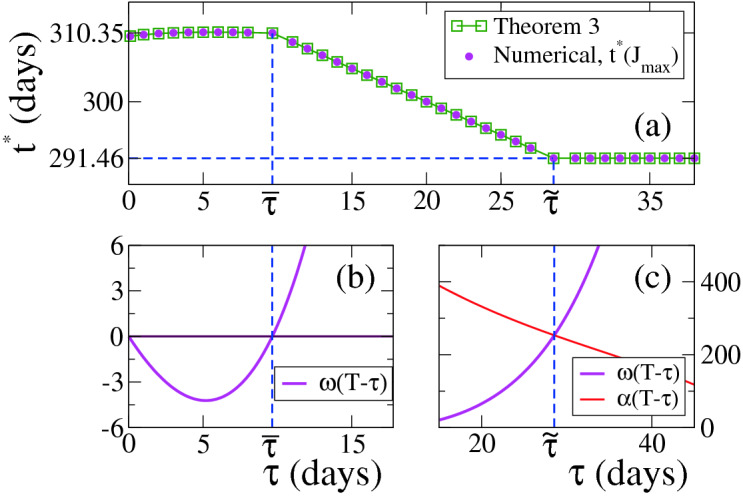
(**a**) Optimal initial time $t^*$ vs strict quarantine length $\tau$ for $\kappa =10^{-5}$ and $w(0)>0$. (**b**) and (**c**) Graphical determination of the times ${\overline{\tau }}$ and ${\tilde{\tau }}$, respectively, which define the three regions for the different behaviours of $t^*$. The parameters are $\gamma =0.1$, $\sigma _f=2.2$, $\sigma _s=0.3$, $\sigma _m=1.5$ and $T=320$. The initial condition corresponds to $x(0)=1-10^{-6}, y(0)=10^{-6}$. The optimum time for the region $\tau > {\tilde{\tau }} \simeq 28.54$ is ${\tilde{t}} \simeq 291.46$, while $t^*$ has a slight dependence on $\tau$ for $\tau \le {\overline{\tau }} \simeq 9.65$.

Given that we consider here $\kappa >0$, *J* reaches a maximum at the optimum time $t^*$ (see Eq. (12)). Figure 7a shows the behaviour of $t^*$ as a function of $\tau$, where we observe a very good agreement between numerical results (circles) and Theorem 3 (squares). We also see that $t^*$ depends slightly on $\tau$ in the $0 \le \tau \le {\overline{\tau }} \simeq 9.65$ interval, while $t^*={\tilde{t}} \simeq 291.46$ for $\tau > {\tilde{\tau }} \simeq 28.54$. The optimal times ${\overline{t}}$ and ${\tilde{t}}$ are estimated as the values of $t=T-\tau$ for which the curve $w(T-\tau )$ crosses the horizontal line 0 and the curve $\alpha (T-\tau )$, respectively (Fig. 7b,c).

**Figure 8:**
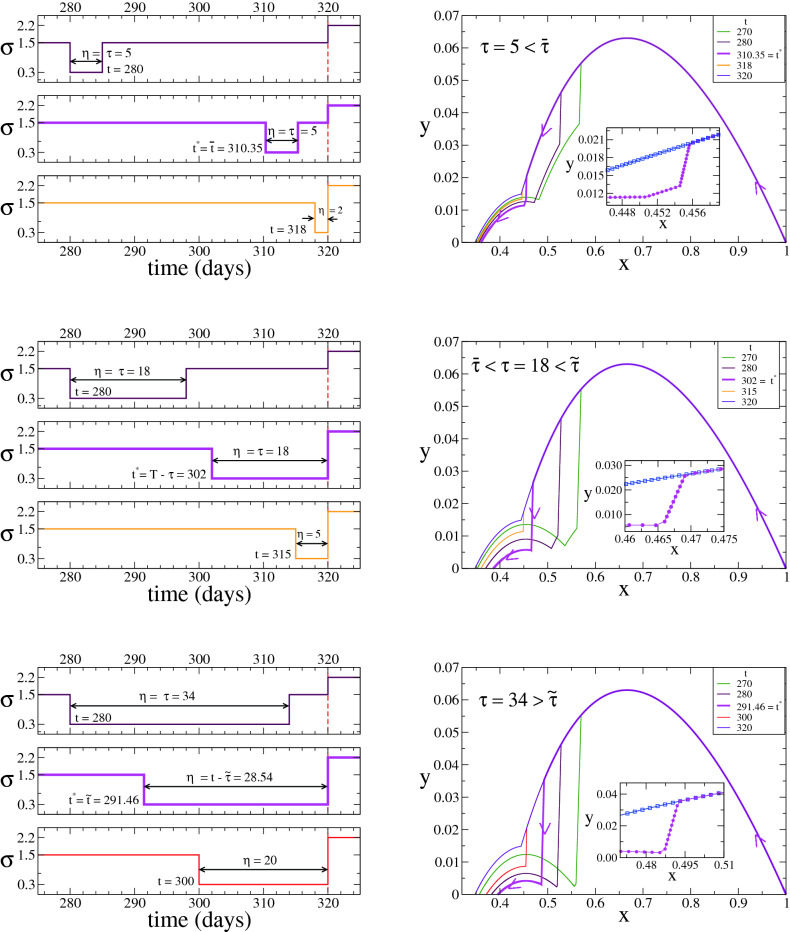
Case $\kappa >0$ and $0< \sigma _s< \sigma _m < \sigma _f$ (Theorem 3). System’s trajectory in the $x-y$ phase space (right panels), for $\gamma =0.1$, $\kappa =10^{-5}$, $\sigma _s=0.3$, $\sigma _m=1.5$, $\sigma _f=2.2$ and $T=320$, and the three values of $\tau$ indicated in the legends corresponding to the different regimes of the optimum time $t^*$ (pink lines). Left panels show the time evolution of $\sigma$ for three different initial times *t* of the strict quarantine in each case. The optimum times are $t^*={\overline{t}} \simeq 310.35$ for $\tau =5$ (top panels), $t^*=T-\tau =302$ for $\tau =18$ (middle panels) and $t^*={\tilde{t}} \simeq 291.46$ for $\tau =34$ (bottom panels).

In the right panels of Fig. 8 we show the system’s evolution in the $x-y$ space for three different values of $\tau$ corresponding to the different behaviour of $t^*$. Unlike the previously studied cases where $\sigma _m=\sigma _f$ (Figs. 4 and 6), here we observe that the curves (*x*(*t*), *y*(*t*)) may exhibit up to three different regimes within the control period *T*, which is due to the fact that $\sigma$ jumps three times in that interval: from $\sigma _m$ to $\sigma _s$ at time *t*, from $\sigma _s$ to $\sigma _m$ at $t+\tau$ and from $\sigma _m$ to $\sigma _f$ at *T*. This can be clearly seen in the $t^*=310.35$ curve for $\tau =5<{\overline{\tau }}$ (inset of top-right panel of Fig. 8). For $\tau$ in the other two regions ($\tau =18$ and 34), the strict quarantine ends at *T* for $t^*$, and thus $\sigma$ jumps twice and (*x*(*t*), *y*(*y*)) exhibits two regimes in [0, *T*] (insets of middle-right and bottom-right panels). As in the previously studied cases, *y*(*t*) drops to the lowest level curve in the interval $[t,t+\eta ]$ for the optimum time $t^*$ (pink curves).

## Discussion and conclusions

In this paper, we have studied an optimal control problem on a SIR dynamics, with a control on the reproduction number $\sigma (t)$ and a limitation in the duration of the intervention *T* and strict quarantine. Based on the Pontryagin’s maximum principle, we have given first order necessary conditions with an overall cost of the epidemic that takes into account both the maximization of the susceptible population in the long term (equivalently, a minimization of the ever infected population) and a penalization of the lockdown associated to a social and economic cost of the epidemic. We also point out that we have employed a novel proof to establish our analytical results. Moreover, some numerical examples have been provided to show the validity of our theoretical results.

Given a fixed time of intervention *T* where control strategies can be applied, and a strict quarantine period $\tau <T$ that represents the maximum time lapse for the stronger intervention, we proved that the optimal strategy is bang-bang when the term representing the socioeconomic cost of the objective functional is linear with respect to the control $\sigma$. More precisely, the optimal solution consists of switching at most twice between a mild ($\sigma =\sigma _m$) and a strict ($\sigma =\sigma _s<\sigma _m$) quarantine, where the latter lasts at most a time period $\tau$.

Although some studies have supported the idea that a too soon or too late intervention may not minimize the total mortality, we found a broader scenario. This is because the optimal solution takes the value $$\sigma =\sigma _s$$ corresponding to the lockdown on an interval $$[t^*,t^*+\eta ]\subseteq [0,T]$$, with $$t^*$$ and $$\eta \le \tau $$ depending on the initial fractions of susceptible and infected individuals $$x_0$$ and $$y_0$$, respectively, and the parameters $$\gamma , \tau , \sigma _f, \sigma _s, \sigma _m$$ and *T*. In fact, we showed that, in some cases, the optimal strategy consists of taking $$t^*= 0 $$ or $$t^*+\eta ^* = T$$ (see Theorem 2 items 1 and 3-4 respectively). However, for an initial condition that corresponds to a real-life case scenario in which the percentage of the population that is infected is small when non-pharmaceutical interventions start, we obtained that the optimal strategy consists on delaying the beginning of the lockdown (items 2-4 from Theorem 2). For the case $$\tau \ll T$$ and $$x_0 < 1/R_0$$, this optimum consists in applying a mild mitigation policy ($$\sigma =\sigma _m$$) at the beginning of the intervention, followed by a strong suppression policy ($$\sigma =\sigma _s$$) and then a mild mitigation again (mild–strict–mild strategy). Here the optimum time $$t^*$$ to start the strict quarantine corresponds to one that leaves the effective reproduction number $$\sigma (t^*+\tau ) x(t^*+\tau )$$ at or just below the threshold value 1.0 when the strict quarantine is released, preventing a new outbreak. For the case $$\tau \lesssim T$$ the optimum corresponds to a mild–strict mitigation strategy, with a strict quarantine that starts late and lasts for a period shorter than $$\tau $$. Surprisingly, it turns more effective to implement a short strict quarantine that starts late than a long strict quarantine that starts early.

We remark that these are optimal strategies within the basic SIR model defined in Eq. (2), which describe in an oversimplified manner the spread of an epidemic on an infinitely large population of individuals with homogeneous recovery, contagion and contact rates, where stochastic fluctuations due to finite-size effects are neglected. Then, stochastic fluctuations in the SIR model on finite populations may play a major role at the beginning of the epidemics if the fraction of infected individuals is relatively small, and thus starting with a strict quarantine may prove more effective if we want to drive the epidemic to extinction. However, we expect that the results presented in this article hold in the limit of very large populations.

We have also studied the possibility of implementing intermittent quarantines, and the possibility of applying suppression measures first, followed by mitigation measures. In both cases, if the total duration of measures is limited, we have shown that they are not optimal in order to maximize the fraction of susceptible individuals at the end of the pandemic.

A major concern with respect to the current COVID-19 crisis is the possibility of an overload of available treatment resources. Since the hospitalized individuals are a fraction of the infected population, a natural objective is to keep the number of infected individuals below some threshold for all times. In a future work we intend to extend our analytic results including a running state constraint that takes this restriction into account. It might also be interesting to study the agent-based version of the SIR model, which naturally accounts for finite-size fluctuations, in order to investigate the role played by stochastic fluctuations in the different optimal strategies described above. It would be worthwhile to explore how the results are affected by the heterogeneity in recovery and infection parameters related to age and social stratum. Finally, we also aim to study the role of an underlying network of contacts, and changes in contact rates due to individual measures triggered by fear of contagion.

## Methods

### Formalization of the optimal control problem

As said in “Results” section, we assume that the function *L* [integrand of $C(\sigma )$] depends linearly on the control $\sigma (t)$. This is a simplified first approach which provides further insight into the structure of the optimal policy. In fact, under this linearity assumption we will prove that the optimal control must be bang–bang (Lemma 2), that is, the strict quarantine is turned *on* and *off*. Thus, we consider that $L(\sigma (t))=\kappa \sigma (t)$ with $\kappa \ge 0$. In this case the functional *J* reads

12 $$\begin{aligned} J(x,y,\sigma )&=x_{\infty }(x(T),y(T),\sigma _f) + \kappa \int _0^T\sigma (t) dt. \end{aligned}$$

Moreover, we consider a restriction on the admissible controls $\sigma$ that assumes that the control can take the value $\sigma _s$ for at most $\tau$ time, and also takes into account a maximum economic cost that the policy maker can afford. In regard to the latter, we consider a maximum cost for imposing the strict lock down for the entire period $\tau$. Smaller values of $\sigma$ represent stricter measures and thus a larger socioeconomical cost. Therefore, we impose an inferior bound to the average of $\sigma$ on [0, *T*], meaning an upper bound for the socioeconomic cost. Thus, we consider the restriction

13 $$\begin{aligned} \int _0^T \sigma ^*(t) dt \ge \sigma _s \tau + \sigma _m (T-\tau ). \end{aligned}$$

Note that any control $\sigma \in [\sigma _s,\sigma _m]$ satisfying Eq. (13) takes the value $\sigma _s$ for a period no longer than $\tau$. In fact, if $\sigma$ is a control that takes the value $\sigma _s$ for a longer period of time than $\tau$, for instance ${\tilde{\tau }}>\tau$, then we would have that $\int _0^T \sigma (t)dt \le \sigma _s {\tilde{\tau }} + \sigma _m(T-{\tilde{\tau }})<\sigma _s \tau + \sigma _m (T-\tau )$ contradicting the inequality from Eq. (13).

We are now in conditions to formalize the problem of finding the optimal control that maximizes the functional *J*. Given $(x_0,y_0)\in {{\mathscr {D}}}$, $T,\tau$ fixed satisfying $0<\tau <T$, $0\le \sigma _s<\sigma _m\le \sigma _f$, we then consider the following optimal control problem with an objective function *J* :

14a $$\begin{aligned} \max \quad&J(x,y,\sigma ):=x_{\infty }(x(T),y(T),\sigma _f) + \int _0^T L(\sigma (t)) dt \end{aligned}$$

14b $$\begin{aligned} \text {s.t.} \quad&x' = -\gamma \sigma (t) x y, \quad x(0) = x_0, \quad t\in [0,T], \end{aligned}$$

14c $$\begin{aligned}y' = \gamma \sigma (t) x y - \gamma y, \quad y(0)= y_0, \quad t\in [0,T], \end{aligned}$$

14d $$\begin{aligned}\sigma _s \le \sigma (t) \le \sigma _m \end{aligned}$$

14e $$\begin{aligned}\int _0^T \sigma (t) dt \ge \sigma _s \tau + \sigma _m (T-\tau ) \end{aligned}$$

In what follows we compute the partial derivatives of $x_{\infty }(x(t),y(t),\sigma )$ with respect to *x*(*t*) and *y*(*t*) in the same way that it is done in^23^. We begin reviewing the solution of the SIR model without control Eq. (1) as done by^23^. It can be shown^1^ that *x*(*t*) satisfies $x(t)e^{\sigma z(t)} = x_0 e^{\sigma z_0}$ which combined with the identity $z(t)=1-x(t)-y(t)$ implies that

$$\begin{aligned} \mu (x(t),y(t),\sigma ):= x(t) e^{-\sigma (x(t)+y(t))} \end{aligned}$$

is constant in time for any solution of Eq. (1). The trajectories in Fig. 1 are also contour lines of $\mu$. Since $y_{\infty }=0$, we then have that $x_{\infty }=x_0 e^{\sigma ( x_{\infty }-x_0-y_0)}=\mu (x_0,y_0,\sigma )e^{\sigma x_{\infty }}$. Then $w=-\sigma x_{\infty }$ satisfies the equation $we^{w}=-\sigma \mu (x_0,y_0,\sigma )$ and therefore $w=W_0(-\sigma \mu (x_0,y_0,\sigma ))$ where $W_0$ is the principal branch of Lambert’s $W-$function^37^, and thus for any $(x,y)\in {{\mathscr {D}}}$

$$\begin{aligned} x_{\infty }(x,y,\sigma )=-\frac{1}{\sigma } W_0(\sigma \mu (x,y,\sigma )). \end{aligned}$$

From this expression we can compute the partial derivatives of $x_{\infty }(x(t),y(t),\sigma )$ with respect to *x*(*t*) and *y*(*t*) in the same way that it is done in^23^.

15a $$\begin{aligned} \frac{\partial x_{\infty }(x(t),y(t),\sigma )}{\partial x(t)}&=\frac{1-\sigma x(t)}{x(t)} \frac{x_{\infty }(x(t),y(t),\sigma )}{1-\sigma x_{\infty }(x(t),y(t),\sigma )}, \end{aligned}$$

15b $$\begin{aligned} \frac{\partial x_{\infty }(x(t),y(t),\sigma )}{\partial y(t)}&=-\frac{\sigma x_{\infty }(x(t),y(t),\sigma )}{1-\sigma x_{\infty }(x(t),y(t),\sigma )}. \end{aligned}$$

In order to solve problem Eq. (14) by means of the Pontryagin’s Maximum Principle, we add a new state variable given by $v(t)=\int _0^t \sigma (s) ds$ and consider $\sigma :[0,T] \rightarrow [\sigma _s,\sigma _m]$ in the class of Lebesgue-measurable functions (so that we have an existence result for optimal solution). Thus, we can study the equivalent optimal control problem:

16a $$\begin{aligned} \max \quad&J(x,y,v,\sigma ):=x_{\infty }(x(T),y(T),\sigma _f) + \int _0^T L(\sigma (t)) dt \end{aligned}$$

16b $$\begin{aligned} s.t. \quad&x'(t)= -\gamma \sigma (t) x(t) y(t), \quad x(0) = x_0, \quad t\in [0,T],\end{aligned}$$

16c $$\begin{aligned}y'(t) = \gamma \sigma (t) x(t) y(t) - \gamma y(t), \quad y(0)= y_0, \quad t\in [0,T],\end{aligned}$$

16d $$\begin{aligned}v'(t) =\sigma (t), \quad v(0)= 0, \quad t\in [0,T], \end{aligned}$$

16e $$\begin{aligned}\sigma (t) \in [\sigma _s,\sigma _m], \quad \text {a.e. } t\in [0,T] \end{aligned}$$

16f $$\begin{aligned}v(T) \ge \sigma _m T +(\sigma _s-\sigma _m) \tau . \end{aligned}$$

We will refer to a 4-tuple $(x,y,v,\sigma )$ as an admissible process of the underlying control system if the control $\sigma$ is a measurable function and the state (*x*, *y*, *v*) is an absolutely continuous vector function satisfying Eqs. (16b)–(16f). The optimal control problem consists in finding an optimal admissible process $(x^*,y^*,v^*,\sigma ^*)$ that maximizes the cost *J*. In this case, we refer to the control $\sigma ^*$ as optimal control.

Next, we give a result on existence of solution for the optimal control problem Eq. (16).

#### **Proposition 1**

*The optimal control problem Eq*. (16) *admits a solution*.

#### *Proof*

Since $L(\sigma )=\kappa \sigma$ is continuous, convex and satisfies that there exists a constant $\alpha _0$ such that for all $\sigma \in [\sigma _s,\sigma _m]$ it holds $L(\sigma )\ge \alpha _0$, the proof follows directly from Theorem 23.11^24^. Using that the control space is closed, solutions of the system of Eqs. (16b), (16c) satisfy that $0\le x+y \le 1$ and the application $x_{\infty }(x,y)$ is continuous, it is straightforward to prove conditions (a) to (f) from Theorem 23.11. Moreover, taking $\sigma (t)\equiv \sigma _m$, we see that the unique solution of the system of Eqs. (16b)–(16d) together with $\sigma$ gives an admissible process for which *J* is finite completing the hypothesis of Theorem 23.11. $\square$

### The optimal control is bang-bang

In what follows, we derive the necessary conditions for problem Eq. (16) where *J* is given by Eq. (12). We consider the Hamiltonian *H*

17 $$\begin{aligned} H(x,y,v,\sigma ,\lambda )= \lambda _0 L(\sigma )-\lambda _1 (\gamma \sigma x y) +\lambda _2 (\gamma \sigma x y -\gamma y ) +\lambda _3 \sigma \end{aligned}$$

where $\lambda _0\ge 0$, $\lambda \in {\mathbb {R}}^3$. The necessary conditions for a maximum process $(x^*,y^*,v^*,\sigma ^*)$ on [0, *T*] are the following^24,25^: There exists a real number $\lambda _0 \ge 0$, the adjoint variable $\lambda :[0,T] \rightarrow {\mathbb {R}}^3$ which is absolutely continuous, and $\beta \in {\mathbb {R}}$ such that $(\lambda _0,\lambda (t),\beta )\ne 0$ for every *t* and the following conditions hold:

1. The adjoint variables $\lambda _1(t),\lambda _2(t)$ satisfy a.e. $t\in [0,T]$
  18a $$\begin{aligned} \lambda _1'(t)&= (\lambda _1(t) -\lambda _2(t)) \gamma \sigma (t) y(t),\end{aligned}$$
  18b $$\begin{aligned} \lambda _2'(t)&= (\lambda _1(t) -\lambda _2(t)) \gamma \sigma (t)x(t)+\gamma \lambda _2(t), \end{aligned}$$
  with final time conditions (using the abbreviation $x_{\infty }$ for $x_{\infty }(x(T),y(T),\sigma _f)$)
  19a $$\begin{aligned} \lambda _1(T)&=\lambda _0 \frac{\partial x_{\infty }}{\partial x(T)}=\lambda _0 \frac{1-\sigma _f x(T)}{x(T)} \frac{x_{\infty }}{1-\sigma _f x_{\infty }}, \end{aligned}$$
  19b $$\begin{aligned} \lambda _2(T)&=\lambda _0 \frac{\partial x_{\infty }}{\partial y(T)}=-\lambda _0\frac{\sigma _f x_{\infty }}{1-\sigma _f x_{\infty }}, \end{aligned}$$
  and the adjoint variable $\lambda _3$ satisfies
  20 $$\begin{aligned} \lambda _3'(t)&=0, \end{aligned}$$
  21 $$\begin{aligned} \lambda _3(T)&=\beta \ge 0 \text { and } \lambda _3(T) (v(T)-\sigma _m (T-\tau ) - \sigma _s \tau )=0 \end{aligned}$$
  obtaining $\lambda _3(t)=\beta$ for all $t\in [0,T]$.
2. For a.e. $$t\in [0,T]$$
  $$\begin{aligned} & \lambda _0 L(\sigma ^*(t)) + \sigma ^*(t) \left( \gamma x^*(t) y^*(t) (\lambda _2(t)-\lambda _1(t))+\beta \right) \\& \quad =\max _{\sigma : \sigma _s\le \sigma \le \sigma _m} \lambda _0 L(\sigma ) + \sigma \left( \gamma x^*(t) y^*(t) (\lambda _2(t)-\lambda _1(t))+\beta \right) . \end{aligned}$$
  Using that $L(\sigma )=\kappa \sigma$ and defining
  22 $$\begin{aligned} \phi (t)=\lambda _0 \kappa +\beta +\gamma x^*(t) y^*(t) (\lambda _2(t)-\lambda _1(t)), \end{aligned}$$
  we obtain for a.e. $t\in [0,T]$,
  23 $$\begin{aligned}\sigma ^*(t) \phi (t) =\max _{\sigma : \sigma _s\le \sigma \le \sigma _m} \sigma \phi (t). \end{aligned}$$
3. There exists a constant *C* such that for a.e. $t\in [0,T]$
  $$\begin{aligned} \lambda _0 L(\sigma ^*(t)) + \sigma ^*(t) \left( \gamma x^*(t) y^*(t) (\lambda _2(t)-\lambda _1(t))+\beta \right) -\gamma \lambda _2(t) y^*(t) =C, \end{aligned}$$
  thus, for all $t\in [0,T]$
  24 $$\begin{aligned} \sigma ^*(t) \phi (t)-\gamma \lambda _2(t) y^*(t) =C. \end{aligned}$$

We have the following result:

#### **Lemma 1**

*The optimal control problem is normal (that is, the multiplier* $\lambda _0\ne 0$*).*

#### *Proof*

Assume $\lambda _0=0$. From Eqs. (18a), (18b) with final time conditions $\lambda _1(T)=\lambda _2(T)=0$, $\lambda _1(t)=\lambda _2(t)=0$ for all $t\in [0,T]$. Since the multipliers $(\lambda _0,\lambda (t),\beta )\ne 0$, then $\lambda _3(t)\equiv \beta >0$ yielding $\phi (t)\equiv \kappa +\beta >0$. Therefore, from the optimality condition given in Eq. (23), $\sigma ^*(t)=\sigma _m$ a.e. $t\in [0,T]$ contradicting the complementarity condition $v(T)=\sigma _m (T-\tau )+\sigma _s \tau$ given in Eq. (21). Thus, we can assume $\lambda _0=1$, and the proof is finished. $\square$

#### **Lemma 2**

*Let* $L(\sigma (t))=\kappa \sigma (t)$ *with* $\kappa \ge 0$ *and let* $\sigma ^*$ *be an optimal control of problem Eq.* (16) *. Then* $\sigma ^*(t)$ *is a bang-bang control.*

#### *Proof*

Assume $\phi (t)=0$ on an interval $[a,b]\subset [0,T]$, then computing its derivative we obtain

$$\begin{aligned} 0=-\gamma x^*(t) y^*(t)\lambda _1(t) \text { for all } t\in (a,b). \end{aligned}$$

Thus, from Eq. (18a) $\lambda _1(t)=\lambda _2(t)=0$ for all $t\in (a,b)$ and therefore for all $t\in [0,T]$, contradicting the end point conditions. Then, there cannot be singular arcs and the control $\sigma ^*$ is given by:

25 $$\sigma ^*(t)= \left\{ \begin{array}{ll} \sigma _m &\text {if }  \; \phi (t)>0\\ \sigma _s&\text {if}   \; \phi (t)<0. \end{array}\right.$$

$$\square $$

#### **Lemma 3**

*Let* $(x_0,y_0)$ *be given and* $(x,y,v,\sigma )$ *an admissible process. Then for* $t\ge 0$

$$\begin{aligned} x_{\infty }(x(t),y(t),\sigma _f)\ge x_{\infty }(x_0,y_0,\sigma _f) , \end{aligned}$$

*and therefore*

26 $$ x_{\infty }(x_0,y_0,\sigma _f) \le x_{\infty }(x(T),y(T),\sigma _f)< 1/ \sigma _f .$$

#### *Proof*

See^23^.

In the next lemma we will see that the switching function changes sign at most two times, concluding that an optimal control $\sigma ^*$ jumps at most twice. $\square$

#### **Lemma 4**

*The switching function* $\phi$ *given in Eq. (*22*) changes sign at most twice.*

#### *Proof*

The proof follows by analysing the phase diagram of $\lambda _1,\lambda _2$. We begin by noting that a solution $(\lambda _1,\lambda _2)$ of the system of Eqs. (18a), (18b) cannot cross both semilines $\lambda _1=\lambda _2>0$ and $\lambda _1=\lambda _2<0$. This is a consequence of condition Eq. (24). In fact, assume there exist $s_1,s_2 \in [0,T]$ such that $\lambda _1(s_1)=\lambda _2(s_1)>0$ and $\lambda _1(s_2)=\lambda _2(s_2)<0$. Evaluating Eq. (22) on $t=s_i$ for $i=1,2$ we have that $\phi (s_i)=\kappa + \lambda _3(T)\ge 0$ and from Eq. (24), $\lambda _2(s_i)=-\frac{C}{\gamma y^*(s_i)}$ if $\kappa +\lambda _3(T)=0$ or, using Eq. (25), $\lambda _2(s_i)=\frac{\sigma _m (\kappa + \lambda _3(T))-C}{\gamma y^*(s_i)}$ if $\kappa +\lambda _3(T)>0$, both cases contradicting that $\lambda _2(s_1)$ and $\lambda _2(s_2)$ had opposite signs.

Since we have end time conditions on *T* we go backwards from $(\lambda _1(T),\lambda _2(T))$ with $\lambda _1(T)>0$ and $\lambda _2(T)<0$. From Eq. (18a), for $\lambda _1<\lambda _2$, $\lambda _1'<0$, thus $\lambda _1$ is decreasing and for the semiplane $\lambda _1>\lambda _2$ we have that $\lambda _1$ is increasing. Also, from Eq. (18b), for $\lambda _1=\lambda _2 >0$ we have that $\lambda _2$ is increasing and for $\lambda _1=\lambda _2 <0$, $\lambda _2$ is decreasing. Finally, from Eqs. (18a) and (18b), for $\lambda _2=0$ and $\lambda _1>0$, both $\lambda _1$ and $\lambda _2$ are increasing.

Thus, since the end time conditions are on the region of the phase diagram with $\lambda _1>\lambda _2$ and $\lambda _2<0$ we have that the solution backwards in time moves to the left where $\lambda _1$ decreases and $\lambda _2$ keeps being negative. At some point in time it could cross the semiline $\lambda _1=\lambda _2<0$ (note that there cannot be touch points). If the solution crosses this line it cannot cross the semiline $\lambda _1=\lambda _2>0$ for a previous time and thus it stays on the region $\lambda _1<\lambda _2$ for all previous times.

From the definition of $\phi$ Eq. (22), for $\lambda _1<\lambda _2$, we have $\phi >0$. For $\lambda _1=\lambda _2$, $\phi =\kappa + \lambda _3(T) \ge 0$ and for $\lambda _1>\lambda _2$, $\phi$ could become negative. Since $\phi '(t)=\gamma x^*(t) y^*(t) \lambda _1(t)$, we see that for $t\in [0,T]$ such that $(\lambda _1(t),\lambda _2(t))$ is on the region $\lambda _1>\lambda _2$ the function $\phi$ decreases for such $t's$ with $\lambda _1(t)<0$ and increases for $\lambda _1(t)>0$. Also, let $s_0,s_1 \in [0,T]$ such that $s_0<s_1$, $\lambda _1(s_0)=\lambda _2(s_0)<0$ and $\lambda _2(s_1)<\lambda _1(s_1)=0$, then $\phi (s_0)=\kappa +\lambda _3(T)$, $\phi$ reaches the minimum value on $[t_0,T]$ at $s_1$ and $\phi (T)=\kappa +\lambda _3(T)-\frac{\gamma y^*(T) x_{\infty }}{1-\sigma _f x_{\infty }} < \phi (s_0)$.

Thus, we conclude that $\phi$ has at most two zeros on [0, *T*] and the proof is finished. $\square$

#### **Theorem 1**

*Let* $(x^*,y^*,v^*,\sigma ^*)$ *be an optimal process, then:*

27 $$\begin{aligned} \sigma ^*(t)= \left\{ \begin{array}{ll} \sigma _m&{} \text {for }0\le t< t_1 \\ \sigma _s &{} \text { for } t_1 \le t < t_1+\eta \\ \sigma _m &{} \text { for } t_1+\eta \le t \le T \end{array}\right. \end{aligned}$$

*with* $$0\le \eta \le \tau $$.

#### *Proof*

Since the optimal control must be bang-bang satisfying Eq. (25), from Lemma 4 it has at most two jumps and from Eq. (13), it takes the value $\sigma _s$ at most for $\tau$ time. Thus the proof is completed.

As a consequence of Theorem 1 we have that if $(x^*,y^*,v^*,\sigma ^*)$ is an optimal process, then the optimal control $\sigma ^*$ is a piecewise constant function having at most two jumps and therefore its unique associated state $(x^*,y^*,v^*)$ is a piecewise continuously differentiable function.

### Characterization of the optimal control

In this section we will give the main Theorem of the article, that characterizes the switching times $t_1$ and $t_1+\eta$ (where $t_1$ is the beginning of the lockdown and $\eta$ is its duration) for an optimal control.

**Figure 9:**
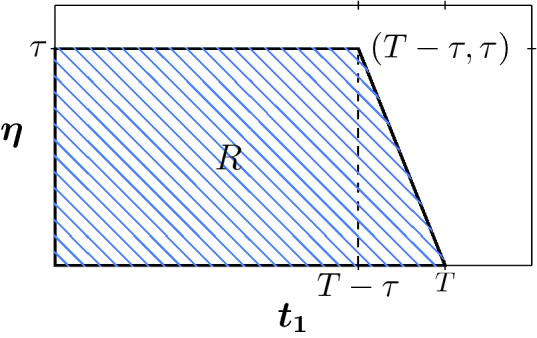
Graphic of set R.

Let us consider the compact set (see Fig. 9)

$$\begin{aligned} R=\left\{ (t_1,\eta ) \in {\mathbb {R}}^2: \,0 \le \eta \le \tau , \,\, 0\le t_1 \le T-\eta \right\} . \end{aligned}$$

Given $(t_1,\eta )\in R$, for simplicity of notation, we will denote $t_0=0, t_2=t_1+\eta$, $t_3=T$.

Moreover, given $(t_1,\eta )\in R$, we will denote the solution of equation Eqs. (16b), (16c) for $s\ge t$ by

28 $$\begin{aligned} \Psi (s,t,x,y,\sigma ), \,\, \text { with } \sigma \in \left\{ \sigma _s,\sigma _m\right\} \,\, \text { and initial data } \,\, (x,y)\in {{\mathscr {D}}} \,\, \text { at time } \, t, \end{aligned}$$

and $(x_1,y_1)=\Psi (t_1,t_0,x_0,y_0,\sigma _m)$, $(x_2,y_2)=\Psi (t_2,t_1,x_1,y_1,\sigma _s)$. Then, if we call $(x_{t_1,\eta },y_{t_1,\eta })$, the solution of equation Eqs. (16b), (16c) associated to the control $\sigma$ given by equation Eq. (27) with initial data $(x(0),y(0))=(x_0,y_0)$, we have that

29 $$\begin{aligned} (x_{t_1,\eta }(s),y_{t_1,\eta }(s))= \left\{ \begin{array}{ll} \Psi (s,t_0,x_0,y_0,\sigma _m)&{} \text {for } 0\le s \le t_1 \\ \Psi (s,t_1,x_1,y_1,\sigma _s) &{} \text { for } t_1< s \le t_2\\ \Psi (s,t_2,x_2,y_2,\sigma _m)&{} \text { for } t_2< s \le T . \end{array}\right. \end{aligned}$$

From Theorem 1 we need to determine the maximum of the function

30 $$\begin{aligned} J(t_1,\eta )=x_{\infty }(x_{t_1,\eta }(T),y_{t_1,\eta }(T),\sigma _f) + \kappa (\sigma _s \eta + \sigma _m (T-\eta )) \end{aligned}$$

on the compact set *R*.

In order to do that, we need to compute the derivatives of *J*.

After some computations (see Eqs. (73) and (76) from Appendix) we obtain that

31 $$\begin{aligned} \frac{\partial J}{\partial t_1} (t_1,\eta )&= \left[ \frac{ \gamma ^2 (\sigma _m-\sigma _s) y_{t_1,\eta }(T) y_{t_1,\eta }(t_1) x_{\infty ,t_1,\eta }}{(1-\sigma _f x_{\infty ,t_1,\eta }) } \right] \nonumber \\&\cdot \left[ \int _{t_1}^{t_2} \frac{\sigma _f x_{t_1,\eta }(r)-1}{y_{t_1,\eta }(r)} dr -\gamma y_2 \int _{t_2}^{T} \frac{\sigma _f x_{t_1,\eta }(r)-1}{y_{t_1,\eta }(r)} dr \int _{t_1}^{t_2} \frac{\sigma _m x_{t_1,\eta }(r)-1}{y_{t_1,\eta }(r)} dr \right] \end{aligned}$$

and

32 $$\begin{aligned} \frac{\partial J}{\partial \eta } (t_1,\eta )=&\frac{\gamma x_{\infty ,t_1,\eta }}{1- \sigma _f x_{\infty ,t_1,\eta }} y_{t_1,\eta }(t_2)(\sigma _m-\sigma _s) \left( 1-(\sigma _f-\sigma _m)y_{t_1,\eta }(T)\gamma \int _{t_2}^T \frac{x_{t_1,\eta }(r)}{y_{t_1,\eta }(r)} dr\right) \nonumber \\&-\kappa (\sigma _m-\sigma _s)\nonumber \\ =&\frac{\gamma x_{\infty ,t_1,\eta }}{1-\sigma _f x_{\infty ,t_1,\eta }} (\sigma _m-\sigma _s)y_{t_1,\eta }(T)\left( 1-\gamma y_{t_1,\eta }(t_2) \int _{t_2}^T \frac{\sigma _f x_{t_1,\eta }(r)-1}{y_{t_1,\eta }(r)} dr\right) \nonumber \\&-\kappa (\sigma _m-\sigma _s) \end{aligned}$$

where $x_{\infty ,t_1,\eta }=x_{\infty }(x_{t_1,\eta }(T),y_{t_1,\eta }(T),\sigma _f)$. Note that for $(t_1,\eta )\in R$, $\frac{\partial J}{\partial \eta } (t_1,\eta )>0$ if and only if

33 $$\begin{aligned} \frac{ x_{\infty ,t_1,\eta }}{1-\sigma _f x_{\infty ,t_1,\eta }}\gamma y_{t_1,\eta }(T)\left( 1-\gamma y_{t_1,\eta }(t_2) \int _{t_2}^T \frac{\sigma _f x_{t_1,\eta }(r)-1}{y_{t_1,\eta }(r)} dr\right) >\kappa . \end{aligned}$$

In the results given in the next sections we will assume that $\frac{\partial J}{\partial \eta }(t_1,\eta )>0$ for all $(t_1,\eta )\in R$ and therefore in the following remark we analyse the derivatives of *J* restricted to the superior border of *R* (see Eq. (37) and (38)) which will be used later.

#### *Remark 2*

Assume that $\frac{\partial J}{\partial \eta }(t_1,\eta )>0$ for all $(t_1,\eta )\in R$, then the maximum value of *J* on *R* must be attained at the superior border

34 $$\begin{aligned} P=\left\{ (t_1,\tau ), \, t_1 \in [0,T-\tau ] \right\} \cup \left\{ (t_1,T-t_1), \, t_1 \in [T-\tau , T] \right\} . \end{aligned}$$

**Figure 10:**
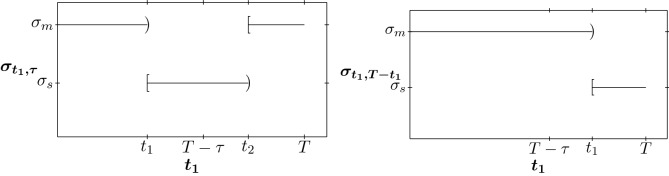
Control for $(t_1,\eta )\in P.$ In (**a**) we have a mild-strict-mild quarantine in the intervention interval [0, *T*]. In (**b**) we have a mild-strict quarantine in the intervention interval [0, *T*].

Thus, in this case, for $(t_1,\tau )$ with $t_1\in [0,T-\tau )$ we have $t_2=t_1+\tau$ and for $(t_1,T-t_1)$ with $t_1\in [T-\tau ,T]$ we have $t_2=T$, and the control is as in Fig. 10

Following, we define a continuous function *w* for $t\in [0,T]$ which is the second factor in equation Eq. (31) and thus gives information on the monotonicity of *J* (see also Eqs. (40) and (41) below).

35 $$\begin{aligned} w(t)= \left\{ \begin{array}{ll} \int _{t}^{t+\tau } \frac{\sigma _f x_{t,\tau }(r)-1}{y_{t,\tau }(r)} dr -\gamma y_{t,\tau }(t+\tau ) \int _{t+\tau }^{T} \frac{\sigma _f x_{t,\tau }(r)-1}{y_{t,\tau }(r)} dr \int _{t}^{t+\tau } \frac{\sigma _m x_{t,\tau }(r)-1}{y_{t,\tau }(r)} dr &{} \text {for }0\le t \le T-\tau \\ &{} \\ \int _{t}^{T} \frac{\sigma _f x_{t,T-t}(r)-1}{y_{t,T-t}(r)}dr &{} \text { for } T-\tau < t \le T, \end{array}\right. \end{aligned}$$

and for $t\in [T-\tau ,T]$, we define

36 $$\begin{aligned} \alpha (t)&= \frac{1}{\gamma y_{t,T-t}(t)}\left( 1-\kappa \frac{1-\sigma _f x_{\infty ,t,T-t}}{ \gamma y_{t,T-t}(T) x_{\infty ,t,T-t} } \right) . \end{aligned}$$

Then we have that for $(t_1,\eta )\in P$

37 $$\begin{aligned} \frac{\partial J}{\partial t_1} (t_1,\eta )&= \frac{ x_{\infty ,t_1,\eta }}{(1-\sigma _f x_{\infty ,t_1,\eta }) } \gamma ^2 (\sigma _m-\sigma _s) y_{t_1,\eta }(T) y_{t_1,\eta }(t_1) w(t_1),\end{aligned}$$

and for $(t_1,T-t_1)\in P$ with $t_1\in [T-\tau ,T]$

38 $$\begin{aligned} \frac{\partial J}{\partial \eta } (t_1,T-t_1)&= \frac{ x_{\infty ,t_1,\eta }}{1-\sigma _f x_{\infty ,t_1,\eta }}\gamma (\sigma _m-\sigma _s)y_{t_1,\eta }(T)-\kappa (\sigma _m-\sigma _s)\nonumber \\&=\frac{ x_{\infty ,t_1,\eta }}{1-\sigma _f x_{\infty ,t_1,\eta }} \gamma ^2(\sigma _m-\sigma _s)y_{t_1,\eta }(T) y_{t_1,\eta }(t_1) \alpha (t_1). \end{aligned}$$

We consider ${\tilde{J}}$ the continuous function defined as the restriction of $J(t_1,\eta )$ to *P*, that is

39 $$\begin{aligned} {\tilde{J}}(t_1)= \left\{ \begin{array}{ll} J(t_1,\tau ) \text { for } t_1\in [0,T-\tau ], \\ &{} \\ J(t_1,T-t_1) \text { for } t_1\in [T-\tau ,T]. \end{array}\right. \end{aligned}$$

From Eq. (37) we have that

40 $$\begin{aligned} {\tilde{J}}' (t_1)&= \gamma ^2 (\sigma _m-\sigma _s) \frac{ x_{\infty ,t_1,\tau }}{(1-\sigma _f x_{\infty ,t_1,\tau })} y_{t_1,\tau }(t_1) y_{t_1,\tau }(T) w(t_1) \quad \text { for } \quad t_1\in [0,T-\tau ), \end{aligned}$$

and from Eqs. (37) and (38), we get

41 $$\begin{aligned} {\tilde{J}}' (t_1)&= \frac{d J}{dt_1} (t_1,T-t_1)-\frac{d J}{d\eta } (t_1,T-t_1) \nonumber \\&= \gamma ^2 (\sigma _m-\sigma _s) \frac{x_{\infty ,t_1,T-t_1}}{1-\sigma _f x_{\infty ,t_1,T-t_1}} y_{t_1,T-t_1}(t_1) y_{t_1,T-t_1}(T) \left( w(t_1)- \alpha (t_1) \right) \end{aligned}$$

for $$t_1\in (T-\tau ,T]$$.

In the next subsection, we prove the main result of this article (Theorem 2) for the case $\sigma _m = \sigma _f$, $\sigma _s\ge 0$ and $\kappa = 0$. Then, we derive the result for $\sigma _s=0$ (Corollary 1) in order to compare our result with the one obtained in^23^.

#### Case $\sigma _m = \sigma _f$ and $\kappa = 0$.

For $\sigma _m=\sigma _f$, from Eq. (74) with $i= 2$, we obtain

42 $$\begin{aligned} w(t) = \begin{aligned} {\left\{ \begin{array}{ll} \dfrac{y_{t,\tau }(t+\tau )}{y_{t,\tau }(T)}\int _{t}^{t+\tau } \frac{\sigma _f x_{t,\tau }(r)-1}{y_{t,\tau }(r)} dr \quad \text { for } 0\le t \le T-\tau \\ \int _{t}^{T} \frac{\sigma _f x_{t,T-t}(r)-1}{y_{t,T-t}(r)} dr \quad \text { for } T-\tau < t \le T. \end{array}\right. } \end{aligned} \end{aligned}$$

In addition, from Eqs. (32) and (74) and using $\kappa =0$ we have that

43 $$\begin{aligned} \frac{\partial J}{\partial \eta } (t_1,\eta )&= \frac{ x_{\infty ,t_1,\eta }}{1-\sigma _f x_{\infty ,t_1,\eta }}\gamma (\sigma _f-\sigma _s)y_{t_1,\eta }(t_1+\eta )>0\end{aligned}$$

for all $$(t_1,\eta )\in R$$.

In this case, the sign and zeros of *w*(*t*) for $t\in [0,T-\tau ]$ are the same as those for the function

44 $$\begin{aligned} z(t)=\int _{t}^{t+\tau } \frac{\sigma _f x_{t,\tau }(r)-1}{y_{t,\tau }(r)} dr, \end{aligned}$$

where *z*(*t*) can be interpreted as the average on the time window $[t,t+\tau ]$ of the difference between the effective reproduction number $\sigma _f x_{t,\tau }(r)$ and the threshold 1.0, weighted by the inverse of $y_{t,\tau }$. Looking at the phase diagram of Fig. 1 we see that the trajectories travel through the contour line $\mu (x_0,y_0,\sigma _m)$ until the strict lockdown is activated, and then descends for the time the lock down lasts (always less than $\tau$) to another contour line of $\mu$ with $\sigma _m$. For $\kappa =0$, the zero of *w*(*t*) ($t*$) captures the moment when this travel to a lower contour line is faster, in the sense that leaves the trajectory on the lowest possible contour line at the end of the strict lockdown and, therefore, at the maximum of $x_{\infty }$. The function *z*(*t*) can also be seen as an external parameter that becomes zero at the optimal time $t^*$ for which *J* reaches its maximum, i.e., $z(t^*)=0$. In the next remark we discuss the sign of *z*(*t*) for $t\in [0,T-\tau ]$ when $\sigma _m=\sigma _f$.

##### *Remark 3*

**Figure 11:**
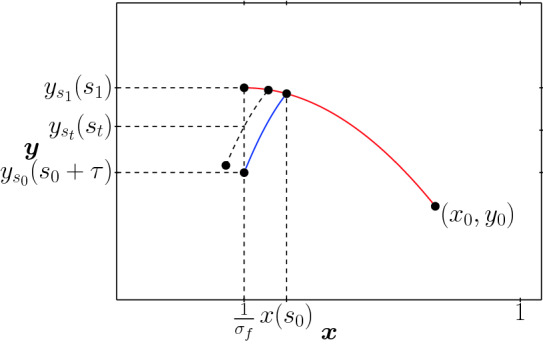
Trajectories $(x_{t,\tau },y_{t,\tau })$ for $t\in [s_0,s_1]$ when $\sigma _m = \sigma _f$.

Given $(x_0,y_0)\in {{\mathscr {D}}}$ with $x_0>1/\sigma _f$, assume there exists $s_1\in [0,T-\tau ]$ such that the solution $\Psi _1(s_1,0,x_0,y_0,\sigma _m)=1/\sigma _f$, that is $x_{s_1,\tau }(s_1)=1/\sigma _f$ (red line in Fig. 11). Then, for $t \in [s_1,T-\tau ]$, $x_{t,\tau }(t)\le 1/\sigma _f$ and therefore $x_{t,\tau }(r)<1/\sigma _f$ for $r\in (t,t+\tau ]$ implying $z(t)<0$. Additionally, assume there exists $s_0\in [0,T-\tau ]$ such that $x_{s_0,\tau }(s_0+\tau )=1/\sigma _f$ (blue line). In this case, it is clear that $s_0<s_1$ and also that for all $t\in (s_0,s_1)$ there exists a unique $s_t \in [t,t+\tau ]$ such that $x_{t,\tau }(s_t)=1/\sigma _f$. Moreover we can conclude that for $t \in [0,s_0]$, $x_{t,\tau }(r)\ge 1/\sigma _f$ for all $r\in (t,t+\tau )$ and therefore $z(t)>0$. If $s_1$ defined before does not exist, that is $x_{s,\tau }(s)>1/\sigma _f$ for all $s\in [0,T-\tau ]$, then we take $s_1=T-\tau$. Likewise, if $s_0$ does not exist, that is for all $s\in [0,T-\tau ]$, $x_{s}(s+\tau )<1/\sigma _f$, then we take $s_0=0$ and conclude that in either case, the sign of *z*(*t*) for $t\ge 0$, is determined in the complement of $[s_0,s_1]$.

In the next lemma we prove that *z* is a decreasing function on the interval $(s_0,s_1)$ introduced in Remark 3.

##### **Lemma 5**

*Let* $s_0,s_1\in [0,T-\tau ]$ *be given by Remark* 3. *Then the function* *z* *defined in Eq.* (44) *is decreasing on* $(s_0,s_1)$*. Moreover,* $z(t)>0$ *for* $t<s_0$, $z(t)<0$ *for* $t>s_1$ *and consequently* *w* *changes sign at most once on *$[0,T-\tau ]$.

##### *Proof*

Since the duration of the strict quarantine is $\tau$ fixed, for simplicity of notation in this proof we neglect the subindex $\tau$ from solutions *x* and *y*. Given $t\in (s_0,s_1)$, we define the auxiliary functions

45a $$\begin{aligned} g_t(s)&=\sigma _f \sigma _s x_t(s) y_t(s)+(\sigma _f x_t(s)-1)(\sigma _s x_t(s)-1), \end{aligned}$$

45b $$\begin{aligned} f_t(s)&=\frac{\sigma _f x_t(s)-1}{y_t(s)}, \end{aligned}$$

45c $$\begin{aligned} i_t(s)&=\frac{x_t(s)}{y_t(s)}(\sigma _f x_t(s)+\sigma _f y_t(s)-1)+\gamma g_t(s) \int _t^s \frac{x_t(r)}{y_t(r)}dr. \end{aligned}$$

By computing the derivative for $s\in (t,t+\tau )$ we obtain

46a $$\begin{aligned} g_t'(s)&=-\gamma \sigma _s^2 x_t(s) y_t(s)(\sigma _f x_t(s)+\sigma _f y_t(s)-1), \end{aligned}$$

46b $$\begin{aligned} f_t'(s)&=-\gamma \frac{g_t(s)}{y_t(s)}, \end{aligned}$$

46c $$\begin{aligned} i_t'(s)&=-\gamma \sigma _s x_t(s)\left( \gamma \sigma _s y_t(s) \int _t^s \frac{x_t(r)}{y_t(r)}dr +1\right) (\sigma _f x_t(s)+\sigma _f y_t(s)-1), \end{aligned}$$

and we have that

47 $$\begin{aligned} z'(t)=&f_t(t+\tau ) - f_t(t) \nonumber \\&+ \gamma (\sigma _s-\sigma _f) y_1 \int _{t}^{t+\tau } \left[ \frac{x_t(s)}{ y_t^2(s)}(\sigma _f x_t(s)+\sigma _f y_t(s)-1) +\gamma \left( \int _{t}^s \frac{x_t(r)}{y_t(r)} dr\right) \frac{g_t(s)}{y_t(s)} \right] ds \nonumber \\ =&f_t(t+\tau ) - f_t(t)+ \gamma (\sigma _s-\sigma _f) y_1 \int _{t}^{t+\tau } \frac{i_t(s)}{ y_t(s)} ds \end{aligned}$$

Note that both $g_t'$ and $i_t'$ have the opposite sign of $(\sigma _f x_t(s)+\sigma _f y_t(s)-1)$. First, assume $\sigma _f x_t(s)+\sigma _f y_t(s)-1>0$ for all $s\in (t,t+\tau )$, then from Eq. (46a), $g_t$ is a decreasing function. Moreover, since $\sigma _s x_t(t+\tau )-1<0$ and $\sigma _f x_t(t+\tau )-1<0$ for $t\in (s_0,s_1)$, we deduce that $g_t(s)>g_t(t+\tau )>0$. In addition, from Eq. (45c) we obtain $i_t$ is positive.

On the other side, from the fact that $x+y$ is a decreasing function, if we assume that there exists $s_3\in [t,t+\tau ]$ such that $\sigma _f x_t(s_3)+\sigma _f y_t(s_3)=1$ we have that for $s<s_3$, $\sigma _f x_t(s)+\sigma _f y_t(s)-1>0$ and for $s>s_3$, $\sigma _f x_t(s)+\sigma _f y_t(s)-1<0$. Therefore, $g_t$ and $i_t$ attains a global minimum on $[t,t+\tau ]$ at $s_3$ and thus $g_t(s)\ge g_t(s_3)=\sigma _f y_t(s_3)>0$ and $i_t(s)\ge i_t(s_3)=\gamma g_t(s_3)\int _{t}^{s_3} \frac{x_t(r)}{y_t(r)} dr>0$ for all $s\in [t,t+\tau ]$ . Furhtermore, from Eq. (46b) we have that $f_t(s)$ is also a decreasing function.

**Figure 12:**
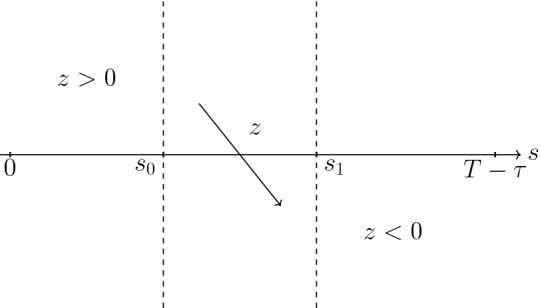
Behaviour of *z*(*t*) when $\sigma _f = \sigma _m$.

Thus, we have proved that for $t\in (s_0,s_1)$, $f_t(s)$ is decreasing on $(t,t+\tau )$ and $i_t(s)>0$ for all $s\in [t,t+\tau ]$, yielding from Eq. (47) that $z'(t)<0$ for all $t\in (s_0,s_1)$. Finally, from Remark 3 we deduce that *z* changes sign at most once on $[0,T-\tau ]$ (see Fig. 12) concluding that *w* changes sign at most once on $[0,T-\tau ]$.

From Eq. (40), we see that the monotonicity of *J* on $[0,T-\tau ]$ depends on the sign of *w*. For all the foregoing, if *J* attains its maximum at $t_1=t^*\in [0,T-\tau ]$, we can interpret *w*(*t*) as an external parameter that shifts the extremal point of *J* from $t^*$ to zero.

In the next theorem we assume that $x_0<1/\sigma _s$. This condition is always satisfied for $\sigma _s<1$. $\square$

##### **Theorem 2**

*Let* $0\le \sigma _s<\sigma _m=\sigma _f$ *with* $\sigma _s<1$, $\kappa = 0$ *and* *w* *be given by Eq*. (42). *Then the optimal control is unique and is given by*

48 $$\begin{aligned} \sigma ^*(s)= \left\{ \begin{array}{ll} \sigma _f&{} \text {for }0\le s<t^*,\\ \sigma _s &{} \text { for } t^* \le s< t^*+\eta ,\\ \sigma _f &{} \text { for } t^*+\eta \le s <T, \end{array}\right. \end{aligned}$$

*where*

1. *For* $$w(0)\le 0$$: $$t^*=0$$ *and* $$\eta =\tau $$.
2. *For* $w(0)>0$ *and* $w(T-\tau )\le 0$: $t^*={\overline{t}}$ *and* $\eta =\tau$ *where* ${\overline{t}}$ *is the unique value on* $[0,T-\tau ]$ *such that* $w({\overline{t}})=0$.
3. *For* $$0<w(T-\tau )\le \dfrac{1}{\gamma y_{T-\tau ,\tau }(T-\tau )}$$: $$t^*=T-\tau $$ *and* $$\eta =\tau $$.
4. *For* $w(T-\tau )>\dfrac{1}{\gamma y_{T-\tau ,\tau }(T-\tau )}$: $t^*={\tilde{t}}$ *where* ${\tilde{t}}$ *is the unique value on* $[T-\tau ,T]$ *such that* $w({\tilde{t}})=\dfrac{1}{\gamma y_{{\tilde{t}},T-{\tilde{t}}}({\tilde{t}})}$ *and* $\eta =T-{\tilde{t}}$.

##### *Proof*

From Eq. (43) and Remark 2 the maximum value of *J* on *R* must be attained at the superior border *P* defined in Eq. (34). Therefore, from Eqs. (40) and (41) we have

49 $$\begin{aligned} {\tilde{J}}' (t_1)&= \gamma ^2 (\sigma _f-\sigma _s) \frac{ x_{\infty ,t_1,\tau }}{(1-\sigma _f x_{\infty ,t_1,\tau })} y_{t_1,\tau }(t_1) y_{t_1,\tau }(T) w(t_1) \quad \text { for } \quad t_1\in [0,T-\tau ), \end{aligned}$$

and using that for $\kappa =0$, $\alpha (t_1)=\dfrac{1}{\gamma y_{t_1,T-t_1}(t_1)}$, then

50 $$\begin{aligned} {\tilde{J}}' (t_1)&= \gamma ^2 (\sigma _f-\sigma _s) \frac{x_{\infty ,t_1,T-t_1}}{1-\sigma _f x_{\infty ,t_1,T-t_1}} y_{t_1,T-t_1}(t_1)y_{t_1,T-t_1}(T) \left( w(t_1)- \dfrac{1}{\gamma y_{t_1,T-t_1}(t_1)} \right) \end{aligned}$$

for $t_1\in (T-\tau ,T]$. Note that from Eq. (75) with $i=1$ and $j=0$, for $t\in [T-\tau ,T]$ it holds the identity

51 $$\begin{aligned} \gamma y_{t_1,T-t_1}(T) \left( w(t_1)- \dfrac{1}{\gamma y_{t_1,T-t_1}(t_1)} \right) = \gamma (\sigma _f-\sigma _s)h(t_1)-1 \end{aligned}$$

where

52 $$\begin{aligned} h(t) = y_{t,T-t}(T)\int _{t}^{T}\frac{ x_{t,T-t}(s)}{y_{t,T-t}(s)}ds \end{aligned}$$

is a decreasing function in $[T-\tau ,T]$. In fact, using that $u_1(s)$ is a positive function [see Eqs. (68)) and (62a)] it is easy to see that

$$\begin{aligned} \frac{d}{dt_1} \left( \frac{y_{t_1}(T)}{y_{t_1}(s)} \right) <0 \end{aligned}$$

and therefore, $h'(t_1) <0$ for $t_1\in (T-\tau ,T)$. From Eqs. (50) and (51) we also have that for $t_1\in [T-\tau ,T]$

53 $$\begin{aligned} {\tilde{J}}' (t_1)&=\gamma (\sigma _f-\sigma _s) \frac{x_{\infty ,t_1,T-t_1}}{1-\sigma _f x_{\infty ,t_1,T-t_1}} y_{t_1,T-t_1}(t_1) \left( \gamma (\sigma _f-\sigma _s)h(t_1)-1\right) \end{aligned}$$

$$\square $$

We consider the following cases:

1. If $w(0)\le 0$, then from Lemma 5, $w(t_1)<0$ for all $t_1 \in (0,T-\tau ]$. Thus, from Eq. (49) we have
  $$\begin{aligned} {\tilde{J}}' (t_1)&\le 0 \text { for all } t_1\in [0,T-\tau ). \end{aligned}$$
  Moreover, using that $w(T-\tau )<0$, the positivity of $\gamma y_{T-\tau ,\tau }(T-\tau )$ and Eq. (51), we obtain that $h(T-\tau )<\dfrac{1}{\gamma (\sigma _f-\sigma _s)}$ and being *h* a decreasing function, from Eq. (53) we deduce that
  $$\begin{aligned} {\tilde{J}}' (t_1)&<0 \text { for all } t_1\in (T-\tau ,T]. \end{aligned}$$
  Therefore $$t^*=0$$.
2. Since $w(0)>0$ and $w(T-\tau )\le 0$, from Lemma 5 there exists an unique ${\overline{t}}\in (0,T-\tau ]$ such that $w({\overline{t}})=0$, $w(t_1)>0$ for all $t_1\in [0,{\overline{t}})$ and $w(t_1)<0$ for all $t_1\in ({\overline{t}},T]$.
  Moreover, since $w(T-\tau )\le 0$, in the same way as for the previous item, we have
  $$\begin{aligned} {\tilde{J}}' (t_1)&<0 \text { for all } t_1\in (T-\tau ,T] \end{aligned}$$
  and from Eq. (49), we obtain
  $$\begin{aligned} {\tilde{J}}' (t_1)&\ge 0 \quad \text { for } t_1<{\overline{t}} \quad \text { and }\quad {\tilde{J}}' (t_1)< 0 \quad \text { for } t_1>{\overline{t}}, \end{aligned}$$
  concluding that $$t^*={\overline{t}}$$.
3. Since
  $$\begin{aligned} 0<w(T-\tau )&<\dfrac{1}{\gamma y_{T-\tau ,\tau }(T-\tau )}, \end{aligned}$$
  from Lemma 5, $w(t_1)>0$ for all $t_1\in [0,T-\tau ]$. On the other side, since $w(T-\tau ) <\dfrac{1}{\gamma y_{T-\tau ,\tau }(T-\tau )}$, from Eq. (52) we have that $h(T-\tau )\le \frac{1}{\gamma (\sigma _f-\sigma _s)}$ and using that *h* is a continuous and decreasing function, we obtain that $h(t_1)<\frac{1}{\gamma (\sigma _f-\sigma _s)}$ for all $t_1\in (T-\tau ,T]$. Thus,
  $$\begin{aligned} {\tilde{J}}' (t_1)> 0 \quad \text { for } t_1\in [0,T-\tau )\quad \text { and } \quad {\tilde{J}}' (t_1)< 0 \quad \text { for } t_1\in (T-\tau ,T]. \end{aligned}$$
  Therefore, $$t^*=T-\tau $$ and $$\eta =\tau $$.
4. Since
  $$\begin{aligned} w(T-\tau )&>\dfrac{1}{\gamma y_{T-\tau ,\tau }(T-\tau )} >0, \end{aligned}$$
  from Lemma 5, we have $w(t_1)>0$ for all $t_1\in [0,T-\tau ]$. On the other side, since $w(T-\tau )>\dfrac{1}{\gamma y_{T-\tau ,\tau }(T-\tau )}$, then $h(T-\tau )>\frac{1}{\gamma (\sigma _f-\sigma _s)}$ and using that $h(T)=0$ and *h* is a continuous and decreasing function, there exists a unique ${\tilde{t}}$ such that $h({\tilde{t}})=\frac{1}{\gamma (\sigma _f-\sigma _s)}$, $h(t)>\frac{1}{\gamma (\sigma _f-\sigma _s)}$ for $t\in [T-\tau ,{\tilde{t}})$ and $h(t)<\frac{1}{\gamma (\sigma _f-\sigma _s)}$ for $t\in ({\tilde{t}},T]$. Therefore,
  $$\begin{aligned} {\tilde{J}}' (t_1)> 0 \quad \text { for } t_1\in [0,{\tilde{t}}) \quad \text { and } \quad {\tilde{J}}' (t_1)< 0 \quad \text { for } t_1\in ({\tilde{t}},T]. \end{aligned}$$
  Consequently, $$t^*={\tilde{t}}$$ and $$\eta = T-{\tilde{t}}$$.

#### Case $\sigma _s= 0$, $\sigma _m = \sigma _f$ and $\kappa = 0$.

Let *w*(*t*) defined as in Eq. (35). Note that for $\sigma _s = 0$ and $\sigma _m= \sigma _f$, from Eq. (75) with $i=j = 2$, we obtain

54 $$\begin{aligned} w(t) = \begin{aligned} {\left\{ \begin{array}{ll} \dfrac{y_{t,\tau }(t+\tau )(\sigma _f x_{t,\tau }(t)-1)}{\gamma y_{t,\tau }(T) y_{t,\tau }(t)}(e^{\gamma \tau }-1) \quad \text { for } 0\le t \le T-\tau \\ \\ \dfrac{\sigma _f x_{t,T-t}(t)-1}{\gamma y_{t,T-t}(t)}(e^{\gamma (T-t)}-1) \quad \text { for } T-\tau < t \le T. \end{array}\right. } \end{aligned} \end{aligned}$$

It is easy to observe that in this case the sign of *w*(*t*) on $[0,T-\tau ]$ is given by $(\sigma _f x_{t,\tau }(t)-1)$. Moreover, *w* changes sign at most once on $[0,T-\tau ]$, going from positive to negative values.

##### **Corollary 1**

*Let* $\sigma _s=0$, $\sigma _m = \sigma _f>0$ *and* $k=0$*. Then, the optimal control is unique and is given by*

$$\begin{aligned} \sigma ^*(s) = \begin{aligned} {\left\{ \begin{array}{ll} \sigma _f \quad \text { for } 0\le s< t^*,\\ 0 \quad \text { for } t^* \le s < t^*+\eta ,\\ \sigma _f \quad \text { for } t^*+\eta \le s \le T, \end{array}\right. } \end{aligned} \end{aligned}$$

*where*

1. *For* $$x_0 \le \frac{1}{\sigma _f}$$ : $$ t^*= 0$$ *and* $$\eta = \tau $$.
2. *For* $x_0 > \frac{1}{\sigma _f}$ *and* $x_{T-\tau ,\tau }(T-\tau ) \le \frac{1}{\sigma _f}$: $t^* = {\overline{t}}$ *and* $\eta = \tau$*, where* ${\overline{t}}$ *is the unique value on* $[0,T-\tau ]$ *such that* $x_{{\overline{t}},\tau }({\overline{t}}) = \dfrac{1}{\sigma _f}$.
3. *For* $$\dfrac{1}{\sigma _f} < x_{T-\tau ,\tau }(T-\tau )\le \dfrac{1}{\sigma _f (1-e^{-\gamma \tau })} $$: $$t^* = T-\tau $$ *and* $$\eta = \tau $$.
4. *For* $x_{T-\tau ,\tau }(T-\tau )> \dfrac{1}{\sigma _f (1-e^{-\gamma \tau })}$: $t^* = {\tilde{t}}$ *and* $\eta = T-{\tilde{t}}$*, where* ${\tilde{t}}$ *is the unique value on* $[T-\tau , T]$ *such that* $x_{{\tilde{t}},T-{\tilde{t}}}({\tilde{t}})= \dfrac{1}{\sigma _f \left( 1-e^{-\gamma (T-{\tilde{t}}) }\right) }$.

##### *Proof*

The proof follows from Theorem 2 using the fact that

$$\begin{aligned} {{\,\mathrm{sign}\,}}(w(t))={{\,\mathrm{sign}\,}}(\sigma _f x_{t,\tau }(t) -1), \quad \text { for } t\in [0,T-\tau ], \end{aligned}$$

and

$$\begin{aligned} w(T-\tau )=\dfrac{1}{\gamma y_{T-\tau ,\tau }(T-\tau )}(\sigma _f x_{T-\tau ,\tau }(T-\tau )-1)(e^{\gamma \tau }-1). \end{aligned}$$

Note that in this case if we take $\tau =T$, the corollary is reduced to only two possible cases: $x_0 \le \dfrac{1}{\sigma _f (1-e^{-\gamma T})}$ or $x_0> \dfrac{1}{\sigma _f (1-e^{-\gamma T})}$, obtaining the same result as Ketcheson^23^ in Theorem 3. $\square$

#### General case

In this section we study the behaviour of optimal solutions for the general case when $0\le \sigma _s<\sigma _m\le \sigma _f$ and $\kappa >0$, that is, objective function *J* includes the term that accounts the running cost of the control and allows us to account for factors like the economic cost of intervention or heightened risks caused by hospital overflow.

##### **Lemma 6**

*Assume* $\kappa > \dfrac{ x_{\infty ,t_1,\eta }\gamma y_{t_1,\eta }(T)}{1-\sigma _f x_{\infty ,t_1,\eta }}\left( 1-\gamma y_2 \int _{t_2}^T \frac{\sigma _f x_{t,\eta }(r)-1}{y_{t,\eta }(r)} dr\right)$ *for all* $(t_1,\eta )\in R$*, then the optimal control is given by* $\sigma ^*\equiv \sigma _m$.

##### *Proof*

From Eq. (32), we have that $\frac{\partial J}{\partial \eta } (t_1,\eta )<0$ and therefore the maximum value of *J* on *R* is attained at the inferior border of *R* where $\eta =0$ and $J(t_1,0)$ is constant.

In the next theorem we give a general result including both the economic cost of intervention ($\kappa >0$) and a mitigation phase different from the no intervention one, that is $\sigma _m<\sigma _f$. In the next section we give numerical simulations supporting this result. When $\sigma =\sigma _f$ and $\kappa =0$ we recover Theorem 2. $\square$

##### **Theorem 3**

*Let* $0\le \sigma _s<\sigma _m\le \sigma _f$ *with* $\sigma _s<1$, $\kappa$ *satisfying Eq.* (33) *for all* $(t_1,\eta )\in R$ *and let* *w* *and* $\alpha$ *defined as in Eqs.* (35) *and * (36) *respectively , then the optimal control is unique and is given by*

55 $$\begin{aligned} \sigma ^*(s)= \left\{ \begin{array}{ll} \sigma _m&{} \text {for }0\le s<t^*,\\ \sigma _s &{} \text { for } t^* \le s< t^*+\eta ,\\ \sigma _m &{} \text { for } t^*+\eta \le s <T, \end{array}\right. \end{aligned}$$

*where*

1. *For* $$w(0)\le 0$$: $$t^*=0$$ *and* $$\eta =\tau $$.
2. *For* $w(0)>0$ *and* $w(T-\tau )\le 0$: $t^*={\overline{t}}$ *and* $\eta =\tau$ *where* ${\overline{t}}$ *is the unique value on* $[0,T-\tau ]$ *such that* $w({\overline{t}})=0$.
3. *For* $$0<w(T-\tau )\le \alpha (T-\tau )$$: $$t^*=T-\tau $$ *and* $$\eta =\tau $$.
4. *For* $w(T-\tau )>\alpha (T-\tau )$: $t^*={\tilde{t}}$ *where* ${\tilde{t}}$ *is the unique value on* $[T-\tau ,T]$ *such that* $w({\tilde{t}})=\alpha ({\tilde{t}})$ *and* $\eta =T-{\tilde{t}}$.

## Data Availability

All relevant data are within the paper.

## Appendix

We begin by computing the derivatives of $x_{t_1,\eta }(T)=\Psi _1(T,t_1+\eta ,x_2,y_2,\sigma _m)$ and $y_{t_1,\eta }(T)=\Psi _2(T,t_1+\eta ,x_2,y_2,\sigma _m)$ with respect to $t_1$.

We recall two properties for the solutions of ordinary differential equations. First, the relation between the derivative with respect to initial time and the derivatives with respect to initial data give us the equation

56 $$\begin{aligned} \frac{\partial \Psi _j(s,t,x,y,\sigma )}{\partial t}&=\frac{\partial \Psi _j(s,t,x,y,\sigma )}{\partial x} \gamma \sigma x y - \frac{\partial \Psi _j(s,t,x,y,\sigma )}{\partial y} \gamma y(\sigma x -1) \end{aligned}$$

for $\Psi$ defined in Eq. (28), with $s\ge t$, $\sigma \in \left\{ \sigma _s,\sigma _m\right\}$, initial data $(x,y)\in \mathscr{D}$ at time *t* and $j=1,2$.

Second, the dependence of the solution $\Psi (s,t,x,y,\sigma )$ with respect to initial data *x*, *y* is given by the following known equations. For simplicity of notation, when there is no risk of confusion, we will denote $\Psi (s)$ for $\Psi (s,t,x,y,\sigma )$,

57 $$\begin{aligned} \left( \begin{array}{ll} \frac{\partial \Psi _1}{\partial x} &{} \frac{\partial \Psi _1}{\partial y} \\ \frac{\partial \Psi _2}{\partial x}&{}\frac{\partial \Psi _2}{\partial y} \end{array}\right) ' (s)= \left( \begin{array}{ll} -\gamma \sigma \Psi _2 (s) &{} -\gamma \sigma \Psi _1(s)\\ \gamma \sigma \Psi _2(s)&{}\gamma ( \sigma \Psi _1(s)-1) \end{array}\right) . \left( \begin{array}{ll} \frac{\partial \Psi _1}{\partial x} &{} \frac{\partial \Psi _1}{\partial y} \\ \frac{\partial \Psi _2}{\partial x}&{}\frac{\partial \Psi _2}{\partial y} \end{array}\right) (s) . \end{aligned}$$

with initial data

58 $$\begin{aligned} \left( \begin{array}{ll} \frac{\partial \Psi _1}{\partial x} &{} \frac{\partial \Psi _1}{\partial y} \\ \frac{\partial \Psi _2}{\partial x}&{}\frac{\partial \Psi _2}{\partial y} \end{array}\right) (t)=Id . \end{aligned}$$

Then we call $\sigma _1=\sigma _s$, $\sigma _2=\sigma _m$ and for $i=1,2$,

59a $$\begin{aligned} u_i(s)=u(s,t_i,x_i,y_i,\sigma _i)=\frac{\partial \Psi _1}{\partial x_i}(s,t_i,x_i,y_i,\sigma _i) - \frac{\partial \Psi _1}{\partial y_i}(s,t_i,x_i,y_i,\sigma _i), \end{aligned}$$

59b $$\begin{aligned} v_i(s)=v(s,t_i,x_i,y_i,\sigma _i)=\frac{\partial \Psi _2}{\partial x_i}(s,t_i,x_i,y_i,\sigma _i) - \frac{\partial \Psi _2}{\partial y_i}(s,t_i,x_i,y_i,\sigma _i), \end{aligned}$$

for $s\in [t_i,t_{i+1}]$, and we have the system of equations on $u_i$ and $v_i$

60 $$\begin{aligned} \left( \begin{array}{l} u_i'(s) \\ v_i'(s) \end{array}\right) = \left( \begin{array}{ll} -\gamma \sigma _i \Psi _2(s) &{} -\gamma \sigma _i \Psi _1(s)\\ \gamma \sigma _i \Psi _2(s)&{}\gamma ( \sigma _i \Psi _1(s)-1) \end{array}\right) . \left( \begin{array}{l} u_i(s) \\ v_i(s) \end{array}\right) . \end{aligned}$$

with initial data

61 $$\begin{aligned} \left( \begin{array}{ll} u_i(t_i)\\ v_i(t_i) \end{array}\right) = \left( \begin{array}{ll} 1\\ -1 \end{array}\right) . \end{aligned}$$

Therefore, after some computations and using Eqs. (56) and (59) we obtain for $s\in (t_1,t_1+\eta )$

62a $$\begin{aligned} \frac{d x_{t_1,\eta }}{dt_1}(s)&= -\gamma (\sigma _m- \sigma _s) x_1 y_1 u_1(s), \end{aligned}$$

62b $$\begin{aligned} \frac{d y_{t_1,\eta }}{dt_1}(s)&=-\gamma (\sigma _m- \sigma _s) x_1 y_1 v_1(s), \end{aligned}$$

and for $$s\in (t_1+\eta ,T]$$

63a $$\begin{aligned} \frac{d x_{t_1,\eta }}{dt_1}(s)&=\gamma (\sigma _m-\sigma _s) x_2 y_2 u_2(s) \nonumber \\&-\gamma (\sigma _m-\sigma _s) x_1 y_1\left( \frac{\partial \Psi _1(s,t_2,x_2,y_2,\sigma _m)}{\partial x_2} u_1(t_2) +\frac{\partial \Psi _1(s,t_2,x_2,y_2,\sigma _m)}{\partial y_2}v_1(t_2)\right) ,\end{aligned}$$

63b $$\begin{aligned} \frac{d y_{t_1,\eta }}{dt_1}(s)&=\gamma (\sigma _m-\sigma _s) x_2 y_2 v_2(s) \nonumber \\&-\gamma (\sigma _m-\sigma _s) x_1 y_1\left( \frac{\partial \Psi _2(s,t_2,x_2,y_2,\sigma _m)}{\partial x_2}u_1(t_2) +\frac{\partial \Psi _2(s,t_2,x_2,y_2,\sigma _m)}{\partial y_2}v_1(t_2) \right) . \end{aligned}$$

Moreover, using that for any $(x_i,y_i)\in {{\mathscr {D}}}$, $\Psi (s,t_i,x_i,y_i,\sigma _i)$, satisfies for $s\in [t_i,t_{i+1}]$

64 $$\begin{aligned} \Psi _1(s,t_i,x_i,y_i,\sigma _i) e^{-\sigma _i (\Psi _1(s,t_i,x_i,y_i,\sigma _i)+\Psi _2(s,t_i,x_i,y_i,\sigma _i))} =x_i e^{-\sigma _i (x_i+y_i)}, \end{aligned}$$

we compute the derivatives with respect to $x_i$ and $y_i$ and using $\Psi _1(s)=\Psi _1(s,t_i,x_i,y_i,\sigma _i)$, we obtain for $s\in [t_i,t_{i+1}]$

65 $$\begin{aligned} \frac{\partial \Psi _1}{\partial x_i}(s)-\sigma _i \Psi _1(s) \left( \frac{\partial \Psi _1}{\partial x_i}(s) +\frac{\partial \Psi _2}{\partial x_i}(s)\right)&=(1-\sigma _i x_i) \frac{\Psi _1(s)}{x_i}, \end{aligned}$$

66 $$\begin{aligned} \frac{\partial \Psi _1}{\partial y_i}(s)-\sigma _i \Psi _1(s) \left( \frac{\partial \Psi _1}{\partial y_i}(s)+\frac{\partial \Psi _2}{\partial y_i}(s)\right)&=-\sigma _i \Psi _1(s). \end{aligned}$$

Then, substracting the last two equations

67 $$\begin{aligned} u_i(s) -\sigma _i \Psi _1(s,t_i,x_i,y_i,\sigma _i) ( u_i(s)+ v_i(s))=\frac{ \Psi _1(s,t_i,x_i,y_i,\sigma _i)}{x_i} \end{aligned}$$

for $s\in [t_i,t_{i+1}]$ and therefore using Eq. (67), from Eq. (60) we have that $u_i$ satisfies the ordinary differential equation

$$\begin{aligned} u_i'(s)&=\gamma \left( \sigma _i \Psi _1(s,t_i,x_i,y_i,\sigma _i)-\sigma _i \Psi _2(s,t_i,x_i,y_i,\sigma _i) -1\right) u_i(s)+\gamma \frac{\Psi _1(s,t_i,x_i,y_i,\sigma _i)}{x_i}, \\ u_i(t_i)&= 1. \end{aligned}$$

In the rest of this section, for simplicity of notation we will denote *x* and *y* for $x_{t_1,\eta }$ and $y_{t_1,\eta }$, defined in Eq. (29), respectively.

Thus, for $s\in [t_i,t_{i+1}]$ when $i=1,2$, we obtain

68 $$\begin{aligned} u_i(s)&=\frac{x(s)y(s)}{x_i y_i}+\gamma \frac{x(s)y(s)}{x_i} \int _{t_i}^{s} \frac{1}{y(r)}dr \nonumber \\&= \frac{x(s)}{x_i }+\gamma \sigma _i \frac{x(s)y(s)}{x_i} \int _{t_i}^{s} \frac{x(r)}{y(r)}dr, \end{aligned}$$

69 $$\begin{aligned} v_i(s)&=-\frac{x(s)}{x_i}+\frac{(1-\sigma _ix(s))y(s)}{x_i}\gamma \int _{t_i}^{s} \frac{x(r)}{y(r)}dr, \end{aligned}$$

70 $$\begin{aligned} u_i(s)+v_i(s)&=\frac{y(s)}{x_i}\gamma \int _{t_i}^{s} \frac{x(r)}{y(r)}dr. \end{aligned}$$

Also, from Eqs. (60) and (66) we can prove for $\Psi (T)=\Psi (T,t_2,x_2,y_2,\sigma _m)$ that

71a $$\begin{aligned} \frac{\partial \Psi _1}{\partial x_2}(T)+\frac{\partial \Psi _2}{\partial x_2}(T) =\frac{y(T)}{y_2}+ (1-\sigma _m x_2)\left( u_2(T)+v_2(T) \right) , \end{aligned}$$

71b $$\begin{aligned} \frac{\partial \Psi _1}{\partial y_2}(T)+\frac{\partial \Psi _2}{\partial y_2}(T) =\frac{y(T)}{y_2}- \sigma _m x_2{x_2} \left( u_2(T)+v_2(T) \right) . \end{aligned}$$

Analogously,

72a $$\begin{aligned} \frac{d x_{t_1,\eta }}{d\eta }(T)&=\gamma (\sigma _m-\sigma _s) x_2 y_2 u_2(T), \end{aligned}$$

72b $$\begin{aligned} \frac{d y_{t_1,\eta }}{d \eta }(T)&=\gamma (\sigma _m-\sigma _s) x_2 y_2 v_2(T) . \end{aligned}$$

We can now compute the derivatives of $J(t_1,\eta )$ given by Eq. (30). From Eq. (15),

73 $$\begin{aligned} \frac{\partial J}{\partial t_1} (t_1,\eta )&= \frac{d x_{\infty }(x_{t_1,\eta }(T),y_{t_1,\eta }(T),\sigma _f)}{d t_1} \nonumber \\&= \frac{ x_{\infty ,t_1,\eta }}{1-\sigma _f x_{\infty ,t_1,\eta }} \left( \frac{1-\sigma _f x_{t_1,\eta }(T)}{x_{t_1,\eta }(T)} \frac{d x_{t_1,\eta }(T)}{d t_1}-\sigma _f \frac{d y_{t_1,\eta }(T)}{d t_1}\right) \nonumber \\&= \frac{ x_{\infty ,t_1,\eta }}{(1-\sigma _f x_{\infty ,t_1,\eta }) x_{t_1,\eta }(T)} \left( (1-\sigma _fx_{t_1,\eta }(T)) \frac{d x_{t_1,\eta }(T)}{d t_1}-\sigma _f x_{t_1,\eta }(T) \frac{d y_{t_1,\eta }(T)}{d t_1}\right) , \end{aligned}$$

and, from Eq. (63)

$$\begin{aligned}(1-\sigma _f x_{t_1,\eta }(T)) \frac{d x_{t_1,\eta }(T)}{d t_1}-\sigma _f x_{t_1,\eta }(T)\frac{d y_{t_1,\eta }(T)}{d t_1}\\&=\gamma (\sigma _m-\sigma _s)x_2y_2 \left( (1-\sigma _f x_{t_1,\eta }(T)) u_2(T)-\sigma _f x_{t_1,\eta }(T)v_2(T) \right) \\&- \gamma (\sigma _m-\sigma _s) x_1 y_1 \left( u_1(t_2) \left( (1-\sigma _f x_{t_1,\eta }(T)) \frac{\partial \Psi _1(T,t_2,x_2,y_2,\sigma _m)}{\partial x_2}+\sigma _f x_{t_1,\eta }(T)\frac{\partial \Psi _2(T,t_2,x_2,y_2,\sigma _m)}{\partial x_2} \right) \right. \\&\left. +v_1(t_2) \left( (1-\sigma _f x_{t_1,\eta }(T))\frac{\partial \Psi _1(T,t_2,x_2,y_2,\sigma _m)}{\partial y_2} +\sigma _f x_{t_1,\eta }(T) \frac{\partial \Psi _2(T,t_2,x_2,y_2,\sigma _m)}{\partial y_2}\right) \right) , \end{aligned}$$

using Eqs. (65), (66), (67) and (71) we obtain

$$\begin{aligned}(1-\sigma _f x_{t_1,\eta }(T)) \frac{d x_{t_1,\eta }(T)}{d t_1}-\sigma _f x_{t_1,\eta }(T)\frac{d y_{t_1,\eta }(T)}{d t_1}\\&=\gamma (\sigma _m-\sigma _s) x_{t_1,\eta }(T)\left[ (1+(\sigma _m-\sigma _f)(u_2(T)+v_2(T))) (y_2-y_1+ (\sigma _m-\sigma _s)x_1y_1 (u_1(t_2)+v_1(t_2)))\right. \\&\left. - (\sigma _m-\sigma _f)x_1y_1\frac{y_{t_1,\eta }(T)}{y_2}(u_1(t_2)+v_1(t_2)) \right] . \end{aligned}$$

Also, using that for $i=1,2$

74 $$\begin{aligned} \frac{1}{y_{i}}-\frac{1}{y_{i+1}}=-\int _{t_i}^{t_{i+1}} \left( \frac{1}{y}\right) '(r) dr=\gamma \int _{t_i}^{t_{i+1}} \frac{\sigma _i x(r)-1}{y(r)} dr, \end{aligned}$$

we have that

75 $$\begin{aligned} y_{i+1}-y_i+\gamma y_i y_{i+1} (\sigma _j-\sigma _i) \int _{t_i}^{t_{i+1}} \frac{x(r)}{y(r)} dr=\gamma y_i y_{i+1} \int _{t_i}^{t_{i+1}} \frac{\sigma _j x(r)-1}{y(r)} dr. \end{aligned}$$

Therefore, from Eqs. (70) and (75)

76 $$\begin{aligned}\frac{(1-\sigma _f x_{t_1,\eta }(T)) \dfrac{d x_{t_1,\eta }(T)}{d t_1}-\sigma _f x_{t_1,\eta }(T)\dfrac{d y_{t_1,\eta }(T)}{d t_1}}{\gamma ^2 (\sigma _m-\sigma _s) x_{t_1,\eta }(T) y_{t_1,\eta }(T)y_1} \nonumber \\&= \left( 1-\gamma y_2 \int _{t_2}^{T} \frac{\sigma _f x(r)-1}{y(r)} dr \right) \int _{t_1}^{t_2} \frac{\sigma _m x(r)-1}{y(r)} dr -(\sigma _m-\sigma _f) \int _{t_1}^{t_2} \frac{x(r)}{y(r)} dr \nonumber \\&= \int _{t_1}^{t_2} \frac{\sigma _f x(r)-1}{y(r)} dr -\gamma y_2 \int _{t_2}^{T} \frac{\sigma _f x(r)-1}{y(r)} dr \int _{t_1}^{t_2} \frac{\sigma _m x(r)-1}{y(r)} dr . \end{aligned}$$

Then, replacing in Eq. (73) we obtain

77 $$\begin{aligned} \frac{\partial J}{\partial t_1} (t_1,\eta )&= \frac{ \gamma ^2 (\sigma _m-\sigma _s) y_{t_1,\eta }(T) y_1 x_{\infty ,t_1,\eta }}{1-\sigma _f x_{\infty ,t_1,\eta }} . \nonumber \\&\left[ \int _{t_1}^{t_2} \frac{\sigma _f x(r)-1}{y(r)} dr -\gamma y_2 \int _{t_2}^{T} \frac{\sigma _f x(r)-1}{y(r)} dr \int _{t_1}^{t_2} \frac{\sigma _m x(r)-1}{y(r)} dr \right] . \end{aligned}$$

Note that for $\sigma _m=\sigma _f$ we have from Eq. (74) for $i=2$ that

$$\begin{aligned} & (1-\sigma _f x_{t_1,\eta }(T)) \frac{d x_{t_1,\eta }(T)}{d t_1}-\sigma _f x_{t_1,\eta }(T)\frac{d y_{t_1,\eta }(T)}{d t_1} \\ & \quad = \gamma ^2 (\sigma _m-\sigma _s) x_{t_1,\eta }(T) y_1y_2 \int _{t_1}^{t_2} \frac{\sigma _m x(r)-1}{y(r)} dr, \end{aligned}$$

and then,

78 $$\begin{aligned} \frac{\partial J}{\partial t_1} (t_1,\eta )&= \frac{ x_{\infty ,t_1,\eta }\gamma ^2 (\sigma _m-\sigma _s) y_1y_2}{1-\sigma _f x_{\infty ,t_1,\eta }} \int _{t_1}^{t_2} \frac{\sigma _m x(r)-1}{y(r)} dr. \end{aligned}$$

On the other hand,

79 $$\begin{aligned} \frac{\partial J}{\partial \eta } (t_1,\eta )&= \frac{d x_{\infty }(x_{t_1,\eta }(T),y_{t_1,\eta }(T),\sigma _f)}{d \eta } -\kappa (\sigma _m-\sigma _s)\nonumber \\&= \frac{ x_{\infty ,t_1,\eta }}{(1-\sigma _f x_{\infty ,t_1,\eta })x_{t_1,\eta }(T)} \left( (1-\sigma _f x_{t_1,\eta }(T)) \frac{d x_{t_1,\eta }(T)}{d \eta }-x_{t_1,\eta }(T)\sigma _f \frac{d y_{t_1,\eta }(T)}{d \eta }\right) -\kappa (\sigma _m-\sigma _s), \end{aligned}$$

where $$x_{\infty ,t,\eta }=x_{\infty }(x_{t,\eta }(T),y_{t,\eta }(T),\sigma _f)$$.

From Eqs. (59a), (59b) and (72), we obtain

80 $$\begin{aligned}\frac{1}{x_{t_1,\eta }(T)}\left( (1-\sigma _f x_{t_1,\eta }(T)) \frac{d x_{t_1,\eta }(T)}{d \eta }-\sigma _f x_{t_1,\eta }(T) \frac{d y_{t_1,\eta }(T)}{d \eta }\right) \nonumber \\&= \gamma y_2(\sigma _m-\sigma _s)\frac{x_2}{x_{t_1,\eta }(T)}\left( (1-\sigma _f x_{t_1,\eta }(T))u_2(T)-\sigma _f x_{t_1,\eta }(T) v_2(T)\right) \nonumber \\&= \gamma y_2(\sigma _m-\sigma _s)\frac{x_2}{x_{t_1,\eta }(T)}\left( (1-\sigma _m x_{t_1,\eta }(T))u_2(T)-\sigma _m x_{t_1,\eta }(T) v_2(T)-(\sigma _f-\sigma _m)x_{t_1,\eta }(T)(u_2(T)+v_2(T))\right) ,\nonumber \\ \end{aligned}$$

and using Eqs. (67), (70) and (75)

81 $$\begin{aligned}= \gamma y_2(\sigma _m-\sigma _s)\frac{x_2}{x_{t_1,\eta }(T)}\left( \frac{x_{t_1,\eta }(T)}{x_2}-(\sigma _f-\sigma _m)x_{t_1,\eta }(T)(u_2(T)+v_2(T))\right) \nonumber \\&= \gamma y_2(\sigma _m-\sigma _s)\frac{x_2}{x_{t_1,\eta }(T)}\left( \frac{x_{t_1,\eta }(T)}{x_2}-(\sigma _f-\sigma _m)x_{t_1,\eta }(T)\frac{y_{t_1,\eta }(T)}{x_2}\gamma \int _{t_2}^T \frac{x(r)}{y(r)} dr\right) \nonumber \\&= \gamma y_2(\sigma _m-\sigma _s)\left( 1-(\sigma _f-\sigma _m)y_{t_1,\eta }(T)\gamma \int _{t_2}^T \frac{x(r)}{y(r)} dr\right) \nonumber \\&= \gamma (\sigma _m-\sigma _s)\left( y_{t_1,\eta }(T)-\gamma y_2 y_{t_1,\eta }(T) \int _{t_2}^T \frac{\sigma _f x(r)-1}{y(r)} dr\right) .\nonumber \\ \end{aligned}$$

Thus, we have

82 $$\begin{aligned} \frac{\partial J}{\partial \eta } (t_1,\eta )&= \frac{ x_{\infty ,t_1,\eta }}{1-\sigma _f x_{\infty ,t_1,\eta }}\gamma (\sigma _m-\sigma _s)y_{t_1,\eta }(T)\left( 1-\gamma y_2\int _{t_2}^T \frac{\sigma _f x(r)-1}{y(r)} dr\right) -\kappa (\sigma _m-\sigma _s).\nonumber \\ \end{aligned}$$
